# Plant volatile-triggered defense in citrus against biotic stressors

**DOI:** 10.3389/fpls.2024.1425364

**Published:** 2024-07-10

**Authors:** Meritxell Pérez-Hedo, Carolina Gallego-Giraldo, María Ángeles Forner-Giner, Raúl Ortells-Fabra, Alberto Urbaneja

**Affiliations:** ^1^ Instituto Valenciano de Investigaciones Agrarias (IVIA), Centro de Protección Vegetal y Biotecnología, Moncada, Valencia, Spain; ^2^ Instituto Valenciano de Investigaciones Agrarias (IVIA), Centro de Citricultura y Producción Vegetal, Moncada, Valencia, Spain

**Keywords:** abiotic stress, antioxidant enzymes, Carrizo citrange, oxidative stress, transcriptome, (Z)-3-hexenyl propanoate

## Abstract

Plants employ sophisticated defense mechanisms, including releasing volatile organic compounds, to defend against biotic and abiotic stresses. These compounds play a crucial role in plant defense by attracting natural enemies and facilitating communication between plants to activate defense mechanisms. However, there has been no research on how exposure to these compounds activates defense mechanisms in citrus plants. To elucidate the underlying mechanisms governing citrus defensive activation, we conducted a molecular analysis of the rootstock Citrange carrizo [a hybrid of *Citrus sinensis* × *Poncirus trifoliata*] in response to defense activation by the volatile (Z)-3-hexenyl propanoate [(Z)-3-HP], utilizing a groundbreaking transcriptomic analysis involving the genomes of both parental species. Our results revealed significant gene expression changes, notably the overexpression of genes related to plant immunity, antioxidant activity, defense against herbivores, and tolerance to abiotic stress. Significantly, *P. trifoliata* contributed most notably to the hybrid’s gene expression profile in response to (Z)-3-HP. Additionally, plants exposed to (Z)-3-HP repelled several citrus pests, attracted natural predators, and led to diminished performance of two key citrus pests. Our study emphasizes the complex molecular basis of volatile-triggered defenses in citrus and highlights the potential of plant volatiles in pest control strategies.

## 1 Introduction

The interaction between plants and herbivores triggers a complex evolutionary battle in which plants have evolved sophisticated defenses to mitigate the effects of herbivory (Kessler and Baldwin, 2002; Howe and Jander, 2008; War et al., 2012). Among these defenses, volatile organic compounds (VOCs) emission plays a pivotal role in plant defense (Dicke, 2009; Dicke and Baldwin, 2010). These compounds not only facilitate complex ecological interactions by attracting natural enemies of herbivores but also act as alarm signals among plants. When neighboring plants detect VOCs, they activate several specific molecular pathways that enhance their defensive capabilities. Among these are the jasmonic acid (JA) and salicylic acid (SA) pathways, which are essential for orchestrating defensive responses (Turlings and Erb, 2018). This underscores the crucial role of VOCs in the intricate dynamics of plant-insect interactions (Frost et al., 2008; Lucas-Barbosa et al., 2011; Kaplan, 2012). Moreover, VOCs pave new pathways for sustainable pest management in agriculture, highlighting the opportunity to explore innovative, effective, and eco-friendly approaches to pest control (Turlings and Erb, 2018).

Recent research has confirmed the potential of VOCs to contribute to sustainable pest and disease control by activating direct and indirect defense mechanisms in plants. The prior exposure of seeds to specific compounds, such as indole, could enhance the resistance of species like *Arabidopsis thaliana* (L.) Heynh (Brassicales: Brassicaceae) against the beet armyworm *Spodoptera exigua* (Hübner) (Lepidoptera: Noctuiddae) and *Medicago truncatula* Gaertn. (Fabales: Fabaceae) against the pea aphid *Acyrthosiphon pisum* (Harris) (Hemiptera: Aphididae) without compromising the vegetative or reproductive development of these plants (Maurya et al., 2022). In tomatoes, research conducted by Yang et al. (2020) demonstrated that the application of (Z)-3-hexenol activated defensive plant responses against the whitefly *Bemisia tabaci* (Gennadius) (Hemiptera: Aleyrodidae). Exposure to this volatile triggered reactions mediated by jasmonic and salicylic acids, thereby increasing the emission of volatile compounds that attracted the parasitoid *Encarsia formosa* (Gahan) (Hymenoptera: Aphelinidae), ultimately enhancing its parasitism capacity on *B. tabaci*. Similarly, exposure to (Z)-3-hexenyl butyrate initiates a series of defense signaling events in tomatoes, including the activation of Ca_2_+ permeable channels, mitogen-activated protein kinases, and the generation of reactive oxygen species through the nicotinamide adenine dinucleotide phosphate (NADPH) oxidase. The effectiveness of exposure to (Z)-3-hexenyl butyrate was previously observed to induce stomatal closure in various plant families such as *Nicotiana*, *Arabidopsis*, *Medicago*, *Zea*, and *Citrus* (López-Gresa et al., 2018), has also been demonstrated in field conditions, resulting in enhanced resistance against infections of *Phytophthora* spp. (Peronosporales: Peronosporaceae) and *Pseudomonas syringae* Van Hall (Pseudomonadales: Pseudomonadaceae) in potato and tomato crops, respectively (Payá et al., 2024). In commercial greenhouse contexts, the application of (Z)-3-hexenyl propanoate [(Z)-3-HP onwards] through slow-release dispensers was shown to reduce the susceptibility of plants to economically important pests such as *Tuta absoluta* (Meyrick) (Lepidoptera: Gelichiidae) in tomatoes (Pérez-Hedo et al., 2021) and *Aulocorthum solani* (Kaltenbach) (Hemiptera: Aphididae) in sweet pepper (Depalo et al., 2022). In tomatoes, the exposition of (Z)-3-HP induced the overexpression of genes associated with anti-herbivore defense, increasing the synthesis of compounds derived from fatty acids, activating the lipoxygenase pathway, and accumulating specific defense compounds (Pérez-Hedo et al., 2021) All these findings converge on a deeper understanding of how Hervivore Induce Plant Volatiles (HIPVs) can be instrumentalized in integrated pest management strategies to activate plant defenses, enhance biological control, and ultimately reduce the dependence on synthetic pesticides in agriculture.

In the context of citrus crops, which hold significant global economic value (Liu et al., 2012; Talón et al., 2020), the substantial threats from various pests and diseases result in considerable losses in both yield and quality (Urbaneja et al., 2020; Wang, 2020). Faced with traditional chemical pest management, which, although effective to a certain extent, often entails environmental and health risks (Tudi et al., 2021), the pressing need to explore more sustainable and environmentally friendly approaches arises (Ahmad et al., 2022). In this scenario, manipulating HIPVs emerges as a novel and promising approach to pest management. To our knowledge, there has been no research on how exposure to VOCs can activate defenses in citrus plants. Citrus plants may have been overlooked in previous research on VOC-induced defenses primarily due to several factors: a historical focus on model plants like *A. thaliana* or tomato, the complexity of citrus physiology and genetics (particularly in hybrid varieties, which present unique challenges such as the genetic variability of hybrid rootstocks and the perennial nature of citrus trees) and the intricate and highly specific interactions within citrus agroecosystems. Consequently, the underlying mechanisms governing this activation in a crop as pivotal as citrus remain elusive.

In this work, the Citrange carrizo (CC) rootstock, a hybrid of *Citrus sinensis* (L.) Osbeck × *Poncirus trifoliata* (L.) Raf. (Sapindales: Rutaceae), is utilized as a model system to examine the molecular responses triggered by exposure to the volatile (Z)-3-HP. This green leaf volatile (GLV) has been shown to activate plant defense mechanisms in other plant species (Pérez-Hedo et al., 2021; Depalo et al., 2022; Riahi et al., 2022). By applying a novel approach that includes transcriptomic analysis using the genomes of both parentals, *C. sinensis*, and *P. trifoliata*, this work aims to decipher how exposure to (Z)-3-HP can modify plant-pest interactions. Furthermore, studying both reference genomes will allow us to determine which parental contributes more to the specific defensive response, highlighting the relevance of one over the other in activating defense mechanisms against herbivores and providing valuable data for future rootstock development programs. Furthermore, considering the increasing importance in recent years of limiting callose accumulation as a result of an over-immunity response in citrus (Hijaz et al., 2020; Ma et al., 2022; Nehela and Killiny, 2023; Sarkar et al., 2024), we biochemically measured whether exposure to (Z)-3-HP influences callose deposition and, consequently, the activity of β-1,3-Glucanase. Lastly, we investigated whether exposure to (Z)-3-HP influences plant selection by pests and natural enemies and if it can reduce infestations by two citrus key pests.

## 2 Materials and methods

### 2.1 Plants, arthropods, and (Z)-3-HP

The plant material utilized in all experiments consisted of the Carrizo citrange (CC) rootstock, a hybrid of *Citrus sinensis* (L.) Osb. and *Poncirus trifoliata* (L.) Raf. CC plants were used when they reached 3 months old and had 8 to 9 fully expanded leaves. The arthropod pests utilized in this study were *Delottococcus aberiae* (De Lotto) (Hemiptera: Pseudococcidae), *Chaetanaphothrips orchidii* (Moulton) (Thysanoptera: Thripidae), *Tetranychus urticae* Koch (Acari: Tetranychidae) and *Aphis spiraecola* Patch (Hemiptera: Aphididae). Eight natural enemies commonly found in citrus crops and associated with the previously described arthropod pests were used in this study. The group comprised the following species: *Aphytis melinus* DeBach (Hymenoptera: Aphelinidae), *Anagyrus vladimiri* Triapitsyn (Hymenoptera: Encyrtidae), *Phytoseiulus persimilis* Athias-Henriot (Acari: Phytoseiidae), *Cryptolaemus montrouzieri* Mulsant (Coleoptera: Coccinellidae), *Adalia bipunctata* (Linnaeus) (Coleoptera: Coccinellidae), *Sphaerophoria rueppellii* (Wiedemann) (Diptera: Syrphidae), *Franklinothrips megalops* Trybom (Thysanoptera: Aeolothripidae) and *Pilophorus clavatus* (L.) (Hemiptera: Miridae). Detailed information on plant growth and arthropod rearing can be found in **Supplementary Methods S1**.

The CC plants chosen for defense activation were placed in a plant growth chamber outfitted with a low-density polyethylene (LDPE) polymer diffuser (Kartell, Fisher Scientific SL, Madrid, Spain) containing 2 ml of the volatile compound (Z)-3-hexenyl propanoate [(Z)-3-HP] (Pérez-Hedo et al., 2021). These plants were exposed to this volatile for 48 hours before use. Control plants were housed in a separate growth chamber under identical conditions: 25 ± 1°C, relative humidity of 60%, and a photoperiod of 14:10 h (L:D), but were not exposed to the elicitor.

### 2.2 RNA isolation and RNA-sequencing

To explore the molecular mechanisms underlying the response of CC plants to (Z)-3-HP, total RNA was isolated from the apical part of nine CC plants exposed to (Z)-3-HP for 48 hours and nine unexposed plants. Both groups were subjected to the same experimental conditions as described above. Each treatment had three biological replicates, with each replicate consisting of pooled RNA from three plants. RNA extraction was performed using the RNeasy^®^ Plant Mini Kit (QIAGEN, Maryland, USA), and genomic DNA removal was carried out using the TURBO DNA-freeTM Kit (Ambion^®^, Life Technologies, CA, USA). RNA integrity was verified via agarose gel electrophoresis and Agilent 2100 Bioanalyzer (Agilent Technologies, Santa Clara, CA, USA), with samples having an RNA integrity number (RIN) of ≥ 7 considered suitable for processing. Library construction and RNA sequencing were performed at the Macrogen NGS service (www.macrogen.com), utilizing the TruSeq Stranded mRNA Library Prep Kit and the Illumina NovaSeq 6000 platform to generate six sequencing libraries employing a 101-base paired-end sequencing approach. **Supplementary Methods S2** describe details about RNA-sequencing, data processing, functional annotation analysis, and DEGs validation by RT-qPCR. Primers used in RT-qPCR validation are listed in **Supplementary Table S1**.

### 2.3 Quantification of β-1,3-glucanase activity

The activity of β-1,3-glucanase was assessed in the apical part of citrus plants using a method based on Miller’s (1959) procedure for measuring reducing sugars from laminarin, as described by Khan & Umar (2021). Frozen plant material was ground, weighed (0.1 g tissue), and homogenized with 1 ml of Sodium Acetate buffer before centrifugation (12000g at 4°C for 10 minutes). The resulting supernatant (50 µL) served as the enzymatic extract, mixed with 50 µL of 0.25% Laminarin Solution. The extract and substrate were mixed and incubated in a thermocycler at 37°C for 10 minutes. After incubation, 100 µL of glucose standards were transferred into 0.25 mL Eppendorf tubes. DNS reagent (0.687% (w/v) 3,5-Dinitrosalicylic acid (DNS), 1.28% (v/v) phenol, 19.92% (w/v) Na-K-tartrate, and 1.226% (w/v) NaOH) (100 µL) was added to each sample, and the mixture was heated at 90°C for 10 minutes, followed by 2 minutes at 25°C before transferring to a microplate for absorbance measurement at 540 nm using a Multiskan SkyHigh Reader (Thermo Scientific, Waltham, MA, USA).

### 2.4 Measuring callose deposition intensity: aniline blue staining and epifluorescence microscopy analysis

Stem samples from citrus plants underwent preparation for microscopic examination aimed at studying callose deposits. Plant material was first fixed in a solution comprising 37% formaldehyde, 100% glacial acetic acid, 95% ethanol, and distilled water in a volumetric ratio of 50:5:10:35 ml and then submerged for two weeks. Following rinsing and sectioning, samples were stained with methyl blue [(1:1) 0.1% methyl blue:potassium phosphate buffer 1M, pH 6.5] for 24 hours and subsequently observed for fluorescence using a Nikon SMZ800N microscope equipped with an epifluorescence system. Callose deposits exhibit bright yellow fluorescence when exposed to UV light. Callose deposit was quantified by measuring the fluorescence area according to the method described by Scalschi et al. (2015). The fluorescent deposits corresponding to stained callose were quantified by analyzing pixel numbers using GIMP (GNU Image Manipulation Program). Five plant sections per treatment were examined, and images were captured utilizing an XM HD995 Nikon digital microscopy camera.

### 2.5 Y-tube bioassays

A Y-shaped olfactometer was employed to evaluate the olfactory preferences of arthropods. Details of the Y-tube used can be found in **Supplementary Methods S3**. A single female individual of each species was introduced into the tube (entry array) and observed until she had walked at least 3 cm up one of the arms or until 15 minutes had elapsed. In the case of *A. spiraecola*, winged females were used. Females who did not choose a side arm within 15 minutes were recorded as ‘no-choice’ and were excluded from data analysis. A total of 40 valid replicates were recorded for each species for each pair of odor sources, except for *T. urticae* and *C. orchidii*, for which 45 replicates were conducted for each, and for *P. clavatus*, where 55 replicates were performed. Each individual was tested only once.

### 2.6 *Delottococcus aberiae* and *Tetranychus urticae* performance

The performance of phytophagous pests, namely the two-spotted spider mite *T. urticae* and the South African mealybug *D. aberiae*, on CC plants exposed to (Z)-3-HP was evaluated in two separate experiments and compared to unexposed plants in each case. Six replicates were conducted for each arthropod pest for each treatment. The method involved placing CC plants in individual entomological cages, each measuring 24.5 × 24.5 × 63 cm (BugDorm-4E2260, MegaView Science Co., Ltd., Taichung, Taiwan). To defensively activate the citrus plants with the Z-(3)-HP volatile, a low-density polyethylene (LDPE) dispenser containing 2 ml of the Z-(3)-HP compound was placed atop the cage (Pérez-Hedo et al., 2021). Control plants were kept in a separate climatic chamber without volatile exposure under identical experimental conditions at 25 ± 2 °C, 60-80% RH, and a 14:10 h (L:D) photoperiod. Forty-eight hours after the dispensers were hung, three N_3_ nymphs of *D. aberiae* were released and placed on the leaves of each plant. Meanwhile, in the experiment with the mite, ten adult females of *T. urticae* were placed on the leaves of each plant. Seven days after the release of the specimens, a weekly count was conducted. The number of female *T. urticae* was assessed in the mite experiment, and the count of nymphs, females, and ovicacs of the mealybug was recorded in the mealybug experiment.

### 2.7 Statistical analyses

Chi-square (*χ* ^2^) goodness-of-fit tests were employed based on a null model to analyze data collected from olfactory responses, including the number of individuals. The odor sources were selected with equal frequency for these *χ* ^2^-tests, which were carried out. Individuals who did not make a choice were excluded from the statistical analysis. The data from the arthropod pests’ performance were analyzed using a Generalized Linear Mixed Model (GLMM) with a Poisson distribution appropriate for the count nature of the data and the structure of the repeated measures. The model included treatment as a fixed effect, while cage and time (weeks) were considered random effects. This approach allowed for the accommodation of intra-cage and intra-temporal correlations. A log link function was utilized to align with the requirements of the Poisson distribution. Mean separation for the number of *D. aberiae* ovisacs per plant was performed using Tukey’s test with a significance level of P < 0.05. All analyses were performed using SPSS version 22.

## 3 Results

### 3.1 RNA-seq analysis: quality and differential expression

Sequencing statistics (**Table 1**) for six RNA-Seq libraries demonstrated the generated data’s quality and quantity. A total of 41,088,056 to 50,843,742 raw reads, corresponding to 7.4 gigabase pairs (Gbp), were generated. After trimming, 40,306,638 to 50,081,708 clean reads (6.2 Gbp) were retained. Low error rates (0.02%) and minimal low-quality reads (0.08-0.13%), along with high Q20 (98.57-98.80%) and Q30 (94.94-95.60%) values, confirmed the high quality of the data.

**Table 1:** Summary of transcriptome mapping and quality statistics.

Samples ID^a^	Raw reads^b^	Read count after trimmed^c^	GC (%)^d^	Q20 (%)^e^	Q30 (%)^f^	Mapped reads *Csi_valencia_1.0* ^g^	Mapped reads *Ptrifoliata_566_v1.3* ^g^	Mapped reads *Csi_valencia_1.0 (%)* ^h^	Mapped reads *Ptrifoliata_566_v1.3 (%)* ^h^
**CC_C1**	49,285,044	47,999,260	44.79	98.57	94.94	38,128,949	42,448,509	79.44	88.44
**CC_C2**	48,904,828	48,047,514	44.45	98.80	95.60	37,961,204	42,496,630	79.01	88.45
**CC_C3**	48,284,922	47,427,916	44.71	98.76	95.50	37,955,438	42,444,133	80.03	89.49
**CC_HP1**	41,088,056	40,306,638	44.25	98.62	95.04	31,323,332	35,277,605	77.71	87.52
**CC_HP2**	50,554,508	49,617,544	44.17	98.70	95.25	39,276,044	44,135,680	79.16	89.95
**CC_HP3**	50,843,742	50,081,708	44.17	98.70	95.33	39,265,233	44,693,930	78.40	89.24

Statistics obtained from HISAT2 the number of cDNA fragments mapped with reference genome Csi_valencia_1.0. and Ptrofoliata_565_v13. Statistic summary of transcriptome sequencing, trimming, assembly and quality scores. ^a^There are three independent biological replicates per treatment. ^b^Raw reads: Total number of reads. ^c^Read count: Total number of cleaned reads after trimming. ^d^GC (%): GC Content. ^e^Q20 (%): Ratio of bases that have phred quality score greater than or equal to 20. ^f^Q30 (%): Ratio of bases that have phred quality score greater than or equal to 30. ^g^Mapped reads: Number of reads mapped according to each reference genome. ^h^Mapped reads percentaje: Percentaje of reads mapped according to each reference genome.

Clean reads were mapped to the reference genomes of *C. sinensis* (Csi_v_1.0) and *P. trifoliata* (Ptrifoliata_565_v1.3) using STAR software, with a maximum intron size of 5 kb, achieving mapping rates of 80.03% and 89.49%, respectively (**Table 1**). A total of 18,944 transcripts from *C. sinensis* and 20,570 from *P. trifoliata* were annotated. After stringent filtering, 2,098 low-quality reads from *C. sinensis* and 2,045 from *P. trifoliata* were removed, resulting in 16,846 and 18,525 expressed genes, respectively, which were subsequently used for data normalization and statistical analysis (**Supplementary Figure S1**). No significant interbatch variability was detected in the normalized data. This was demonstrated by Pearson’s correlation coefficients ranging from 0.9 to 1.0 in the correlation matrix (**Supplementary Figure S2A**), indicating high similarity between the data sets across treatments. Additionally, both hierarchical clustering (**Supplementary Figure S2B**) and multidimensional scaling (MDS) analyses (**Supplementary Figure S2C**) shown similar expression patterns between sample groups, confirming their suitability for further study.

**Figure 1:**
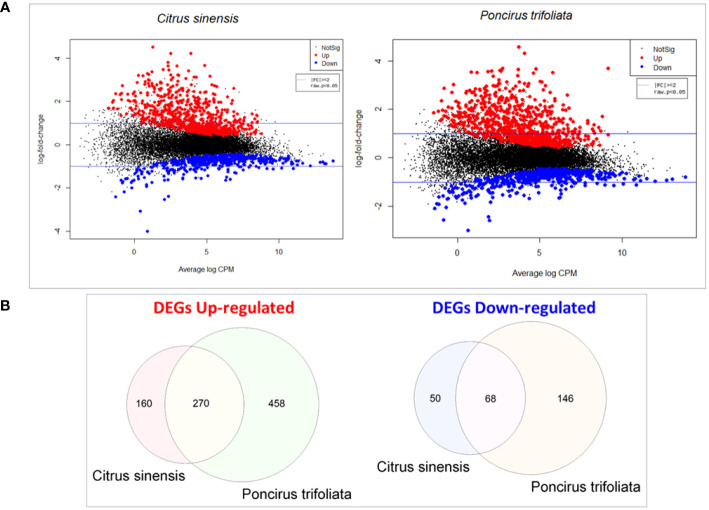
Comparative gene expression analysis in *Citrus sinensis* and *Poncirus trifoliata* exposed to (Z)-3-HP: **(A)** Smear plots were generated to illustrate the most significant changes in gene expression levels for both *C.sinensis* and *P.trifoliata* when exposed to (Z)-3-HP. The x-axis represents the Average log CPM (Counts Per Millon), that indicates the overall average expression level of the genes. The y-axis represents log2 Fold Change that denotes the ratio of expression levels between the two experimental conditions. Positive values signify upregulation, while negative values indicate downregulation. The log2 transformation allows for a symmetric comparison of upregulation and downregulation. **(B)** Veen diagrams were constructed to visualize the overlap and differences in differentially expressed genes (DEGs) between *C.sinensis* and *P.trifoliata* genomes.

Transcripts that were expressed in pairwise comparisons between (Z)-3-HP-exposed and control plants, with a raw p-value < 0.05 and a fold change (│FC│) threshold of ≤ -2 or ≥ 2, were classified as differentially expressed genes (DEGs) (**Figure 1A**). We identified 548 DEGs in *C. sinensis* and 942 in *P. trifoliata*, of which 430 C*. sinensis* and 728 P*. trifoliata* transcripts showing upregulation. In contrast, 118 C*. sinensis* and 214 P*. trifoliata* transcripts were downregulated. A comparison of the two transcriptomes revealed 270 common upregulated genes and 68 common downregulated genes (**Figure 1B**). This indicates that a significant proportion of 458 upregulated and 146 downregulated DEGs in *P. trifoliata* (more than 50%) were not shared with *C. sinensis*.

**Figure 2:**
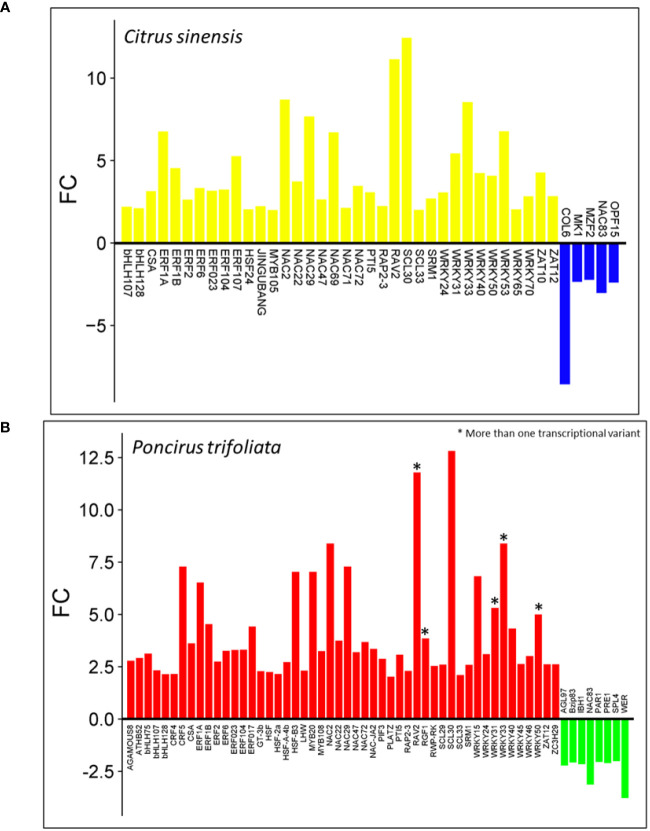
List of transcription factors (TFs) differentially expressed in response to (Z)-3-HP exposure: **(A)** *Citrus sinensis*: Upregulated TFs are highlighted in yellow. Downregulated TFs are highlighted in blue. **(B)** *Poncirus trifoliata*: Upregulated TFs are indicated in red. Downregulated TFs are indicated in green. An asterisk (*) denotes genes with multiple transcriptional variants.

Most of the differentially expressed genes (DEGs) encode proteins, with 94.5% in *C. sinensis* and 90.5% in *P. trifoliata*. Among these protein-encoding transcripts, we identified 36 upregulated and 5 downregulated transcription factors (TFs) in *C. sinensis* (**Figure 2A**). In contrast, *P. trifoliata* exhibited 50 upregulated and 8 downregulated TFs, some of which have transcriptional variants (**Figure 2B**). Notably, 27 upregulated TFs are shared between the two transcriptomes, while no downregulated TFs are shared. This underscores the significant role of TFs in the differential gene expression profiles observed in both genomes. Within this group, several transcription factor (TF) families play critical roles in plant immune responses and hormonal signaling crosstalk, including WRKY, ERF, HSF, PTI5, and CRF. The CRF family is essential for the regulation of cytokinin-responsive genes and is unique to the *P. trifoliata* genome. Other families identified, such as bHLH and MYB, are crucial for secondary metabolite biosynthesis and hormone signaling. In addition, TF families involved in abiotic stress response and the abscisic acid (ABA) signaling pathway were identified, including ZAT, PLATZ, HD-Zip, and RAV. Furthermore, TF families such as NAC, SCARECROW-like, and MADS-box, which are required for plant physiological development, were identified, with the MADS-box family being exclusive to the *P. trifoliata* genome (**Figures 2A, B**). This diverse representation of TFs underline the transcriptional reprogramming that occurs in CC plants in response to (Z)-3-HP cues derived from their genetic background.

We identified 30 transcripts in *C. sinensis* and 146 transcripts in *P. trifoliata* that do not encode proteins, revealing the dynamic genomic landscape of CC in response to the (Z)-3-HP stimulus. Among these, we found 7 long non-coding RNAs (lncRNAs) in *C. sinensis* and 63 P*. trifoliata*, 2 small nucleolar RNAs (snoRNAs) in *C. sinensis* and 8 in *P. trifoliata*, 2 microRNAs (miRNAs) in *C. sinensis* and 3 in *P. trifoliata*, 2 ribosomal (rRNAs) in *C. sinensis* and 8 in *P. trifoliata*, and 2 transfer RNAs (tRNAs) in *P. trifoliata*, as well as 19 pseudogenes in *C. sinensis* and 54 in *P. Trifoliata*.

To validate the differentially expressed genes (DEGs) identified through RNA sequencing, a subset of 20 DEGs was randomly selected for RT-qPCR analysis using the same samples used for RNA-seq analysis. The results of RT-qPCR demonstrate a strong positive correlation with the RNA-seq data, as evidenced by Pearson correlation coefficients of 0.97387 and 0.97435, using GAPH and EF1 as internal controls, respectively (**Supplementary Figure S3**). These findings confirm the validity and reliability of the RNA-seq results.

### 3.2 Gene ontology and pathway analysis

Our study identified 235 upregulated DEGs in *C. sinensis* and 425 upregulated DEGs in *P. trifoliata*, along with 30 downregulated DEGs in *C. sinensis* and 44 downregulated DEGs in *P. trifoliata*, which were associated with various Gene Ontology (GO) categories and Kyoto Encyclopedia of Genes and Genomes (KEGG) pathways.

The analysis identified 35 upregulated and 18 downregulated Gene Ontology (GO) categories in *C. sinensis*, and 45 upregulated and 18 downregulated GO categories in *P. trifoliata*. Among them, 23 upregulated and 16 downregulated GO categories in *C. sinensis*, and 36 upregulated and 16 downregulated GO categories in *P. trifoliata* were statistically significant. Additionally, we assigned 6 upregulated and 1 downregulated KEGG category in *C. sinensis*, and 8 upregulated and 5 downregulated KEGG categories in *P. trifoliata*, all of which were statistically significant.

**Figure 3:**
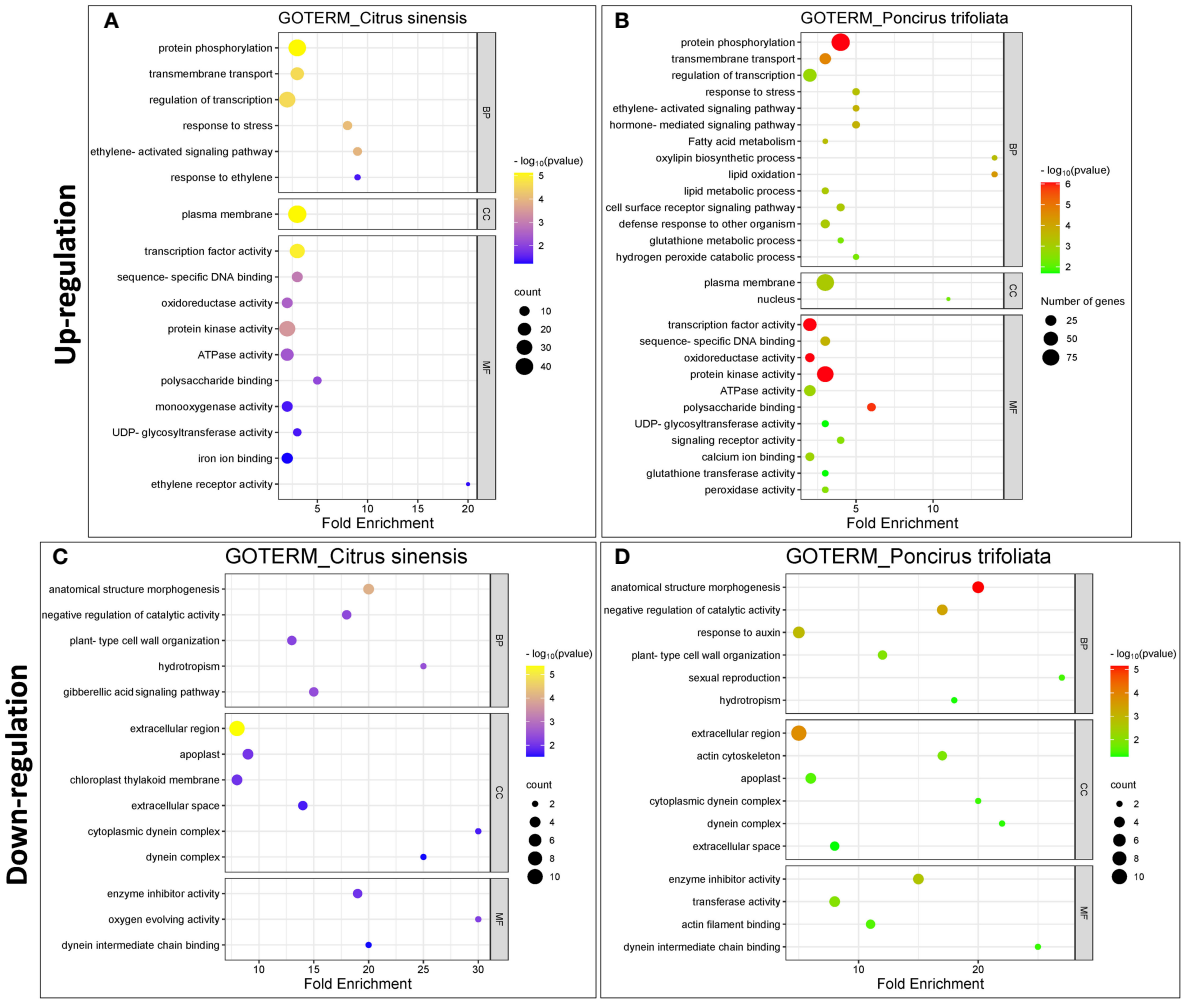
Enriched Gene Ontology (GO) terms in response of CC plants to (Z)-3-HP exposure in *Citrus sinensis* and *Poncirus trifoliata*. **(A, B)** Upregulated GO terms for *C.sinensis* and *P.trifoliata*, respectively. **(C, D)** Downregulated GO terms for *C.sinensis* and *P.trifoliata*, respectively. Categories include: Biological Process (BP), Molecular Function (MF), and Cellular Component (CC). The color scale ranges from yellow to blue for *C.sinensis* and from red to green for *P.trifoliata*, indicating the p-value (-log10). The x-axis represents the fold enrichment values for each GO term. The size of the geometric circles reflects to the number of genes associated with each category.

In the upregulated DEGs, we focused on 19 enriched GO functional domains within the molecular function category (**Figures 3A, B**). Notable shared functions between *C.sinensis* and *P.trifoliata* included “transcription factor activity” (GO:0003700), “protein kinase activity” (GO:0004672), “ATPase activity” (GO:0016887), “oxidoreductase activity” (GO:0016491) and “polysaccharide binding” (GO:0030247). Unique to *Citrus sinensis* were functions such as “ethylene receptor activity” (GO:0038199), “monooxygenase activity” (GO:0004497) and “iron ion binding” (GO:0005506). In contrast, *P. trifoliata* showed exclusive activities such as “peroxidase activity” (GO:0004601), “glutathione transferase activity” (GO:0004364), “calcium ion binding” (GO:0005509), and “signaling receptor activity” (GO:0038023). These results suggest that *P.trifoliata* plays a pivotal role in conferring adaptive stress responses, including the regulation of redox balance, to the hybrid progeny. In the GO analysis of the downregulated DEGs within the molecular function category (**Figures 3C, D**), the prominent common terms were “enzyme inhibitor activity” (GO:0004857), along with “dynein intermediate chain binding” (GO:0045505). The downregulated terms “transferase activity” (GO:0016740) and “actin filament binding” (GO:0051015) were exclusively present in the *P. trifoliata* genome.

In our exploration of biological processes among upregulated DEGs, we identified several common processes in both genetic backgrounds. These included the “ethylene-activated signaling pathway” (GO:009873), “response to stress” (GO:0033554), “regulation of transcription” (GO:0006357), “transmembrane transport” (GO:0055085), and “protein phosphorylation” (GO:0006468) (**Figures 3A, B**). The GO term “response to ethylene” (GO:0009723) was exclusively observed in *C. sinensis*, indicating its significant role in ethylene-mediated responses. Conversely, antioxidant-related events such as “hydrogen peroxide catabolic processes” (GO:0042744) and “glutathione metabolic process” (GO:0006749) were uniquely identified in the *P. trifoliata* genome. Furthermore, the activation of hormonal pathways associated with defense responses was exclusive to the *P. trifoliata* genome. This includes processes such as “defense response to other organism” (GO:0098542), “hormone-mediated signaling pathway” (GO:0009755), “fatty acid metabolic processes” (GO:0006631), “oxylipin biosynthesis” (GO:0031408), and “lipid oxidation” (GO:0034440). These findings highlight the potential role of *P. trifoliata* in conferring enhanced stress responses and metabolic regulation, contributing to CC adaptive resilience. Downregulated biological process domains (**Figures 3C, D**) included common terms in both genetic backgrounds, such as “negative regulation of catalytic activity” (GO:0043086), “plant-type cell wall organization” (GO:0009664), “hydrotropism” (GO:0010274), and “anatomical structure morphogenesis” (GO:0009653). The “gibberellic signaling pathway” (GO:0010476) was exclusive to *C.sinensis*, while “response to auxin” (GO:0009733) was unique to *P.trifoliata*.

In terms of cellular component (CC) categories, upregulated DEGs in both backgrounds were mainly localized to the “plasma membrane” (GO:0005886) (**Figures 3A, B**). Notably, *P.trifoliata* exclusively showed genes with nuclear localization, specifically in the “nucleus” (GO:0005634). Downregulated DEGs were functionally enriched in the terms “extracellular region” (GO:0005576), “extracellular space” (GO:0005615), “apoplast” (GO:0048046), and “dynein complex” (GO:0030286) for both genetic backgrounds (**Figures 3C, D**). Interesting the term “chloroplast thylakoid membrane” (GO:0009535) was unique in *C.sinensis* background.

**Figure 4:**
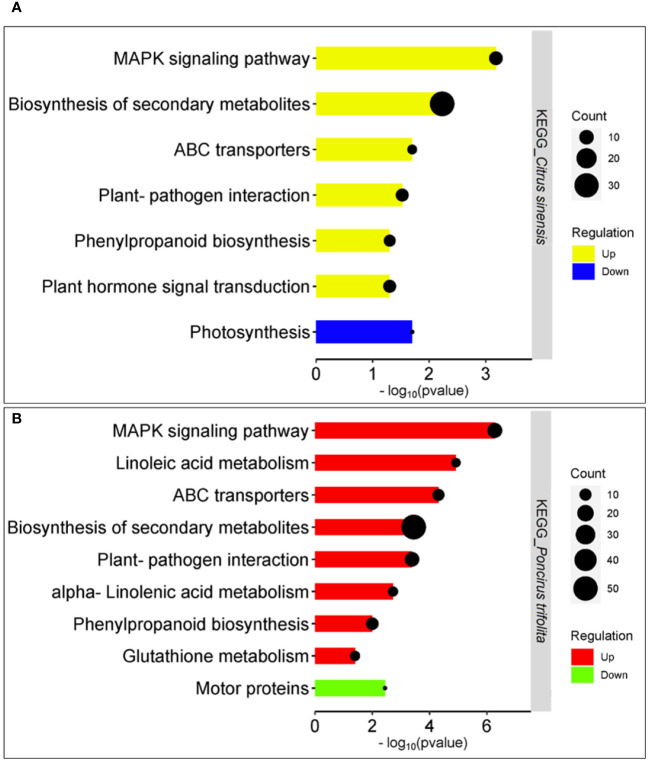
Enriched KEGG pathways in response of CC plants to (Z)-3-HP exposure in *Citrus sinensis* and *Poncirus trifoliata*. **(A)** *Citrus sinensis*: Upregulated pathways are highlighted in yellow, while downregulated pathways are highlighted in blue. **(B)** *Poncirus trifoliata*: Upregulated pathways are indicated in red, and downregulated pathways are indicated in green. The x-axis represents the p-value (-log_10_) obtained for each KEGG pathway. The size of the geometric circles corresponds to the number of genes associated with each KEGG pathway.

In the context of upregulated KEGG pathways, the “MAPK signaling pathway” (cit04016) and “Biosynthesis of secondary metabolites” (cit0110) exhibited the highest statistical significance (*P* < 0.001) in both backgrounds (**Figures 4A, B**), with the latter containing the most significant number of DEGs (31 DEGs in *C.sinensis* and 51 DEGs in *P.trifoliata*). Additionally, exclusive pathways enrichment were noted in the *P. trifoliata* genome (**Figure 4B**), including “Linoleic acid metabolism” (cit00591), “ABC transporters” (cit02010), “Plant-pathogen interaction” (cit04626), and “alpha-Linoleic acid metabolism” (cit00592), all demonstrating significantly high enrichment (*P* < 0.001). A common pathway “Phenylpropanoid biosynthesis pathway” (cit00940), was detected with statistical significance of *P* < 0.05 for *C.sinensis* and *P* < 0.01 for *P.trifoliata*. Additionally, the pathway “Plant hormone signal transduction” (cit:04075) (*P* < 0.05) was unique to *C.sinensis*, while “Glutathione metabolism” (cit:00480) (*P* < 0.05) was exclusive for *P.trifoliata*. In contrast, the only downregulated KEEG pathway detected in *C.sinensis* was “photosynthesis” (cit00195) (**Figure 4A**), while in *P.trifoliata*, the downregulated pathway was “Motor proteins” (cit:04814) (**Figure 4B**), both with a significance range of *P* < 0.05.

### 3.3 Identification of key genes and pathways through systems-level analysis

**Figure 5:**
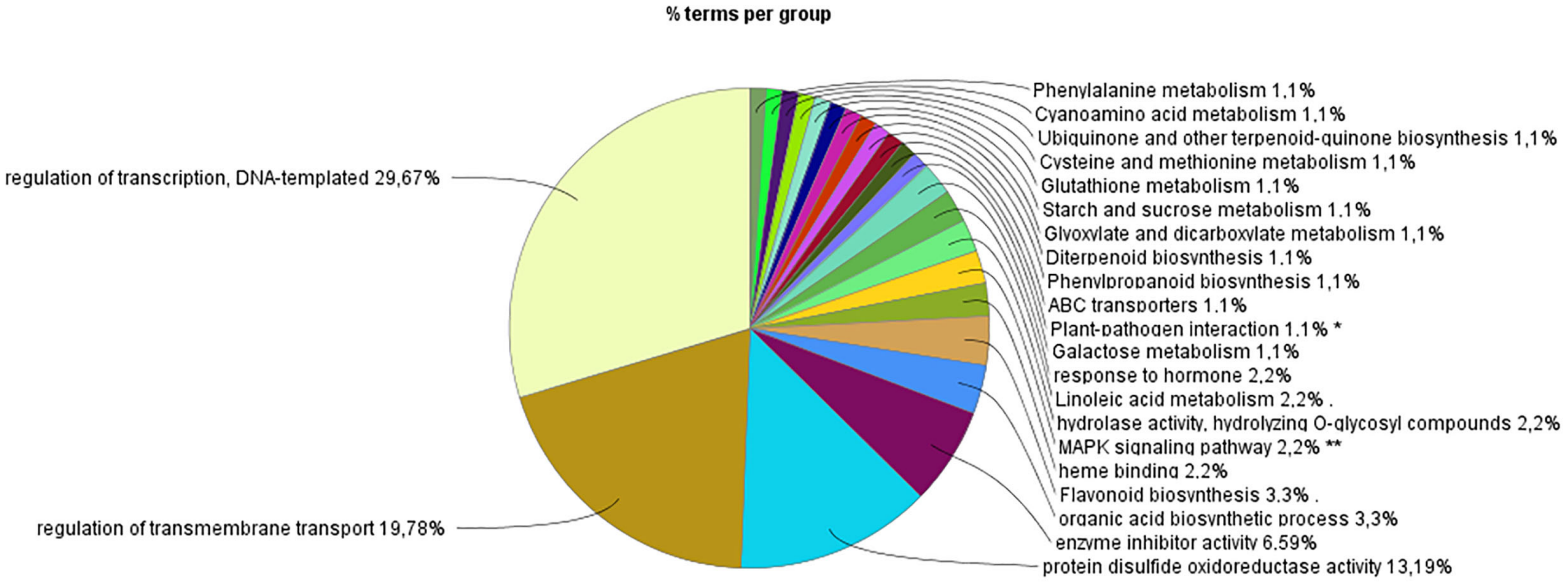
Gene set enrichment analysis (GSEA) of pathways associated with volatile compound induction in citrus. Pie chart illustrating the overall distribution of enriched pathways, categorized into five broad functional groups. The top 4 represent 69.23% of the total functional enrichment.

**Figure 6:**
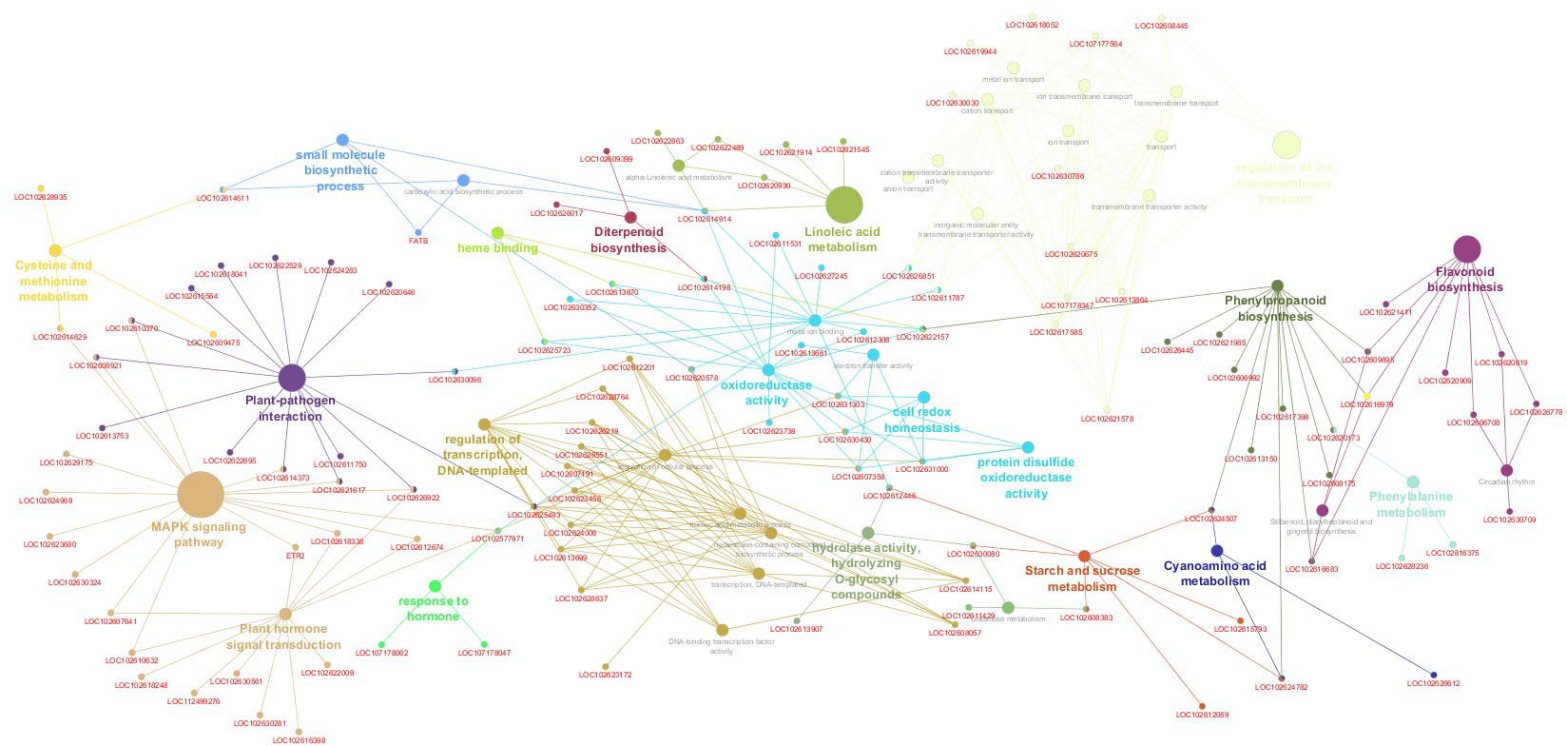
Network visualization of pathways enriched from gene set enrichment analysis (GSEA) performed on a citrus volatile induction. Each node in the network represents a pathway, with its size proportional to the enrichment score, indicating their hierarchies, relationships, and key gene sets within each pathway.

The Gene Set Enrichment Analysis (GSEA) revealed that approximately 50% of the enriched DEGs were linked to pathways regulating transcription and transmembrane transport. In comparison, the remaining 50% were associated with plant defense and developmental pathways (**Figure 5**). Specifically, 13% were associated with oxidoreductase activity, 6.59% with inhibited enzyme activity, and 24% with terms related to the plant immune system, hormone biosynthesis and response, secondary metabolite production, and cell wall development. The graphical representation of enriched functional terms, their connections, and associated gene sets (**Figure 6**) highlights critical pathways and candidate genes likely to play pivotal roles in the studied biological context. This information lets us identify primary gene clusters associated with functional modules involved in specific biological responses within our experimental framework.

### 3.4 MAPK signaling pathways and antioxidant response

The enrichment of MAPKs (Mitogen-Activated Protein Kinases) is noteworthy, representing 2.2% of functional DEGs. This gene-set includes 9 and 16 upregulated genes in *C.sinensis* and *P.trifoliata*, respectively. One of the main functions of MAPKs is the phosphorylation of specific target proteins in signaling cascades, leading to coordinated cellular and physiological responses. Correlated with the higher functional enrichment in the GOterm category “protein phosphorylation” observed in the two genomes. One of the most outstanding biological processes of this type of kinase is the modulation of H_2_O_2_ levels and restoring redox balance by activating the antioxidant system. Notable genes within this pathway include *1-aminocyclopropane-1-carboxylate synthase* (*LOC102614629*), *EIN3* (*LOC102607641*), *WRKY24* (*LOC102621617*), *Endochitinase* (*LOC102623680*), *ETR2*, *ETR1* (*LOC102577971*), *MAPK9* (*LOC102608921*), *WRKY33* (*LOC102608921*), *CML46* (*LOC102616804*), *LOC102629175*, *Receptor kinase* (*LOC102622580*), *RBOHB* (*LOC102610370*), *SAPK3* (*LOC102622961*), *SRK2E* (*LOC102612674*), *SRK2I* (*LOC102610632*), and *VIP1* (*LOC102630324*).

**Figure 7:**
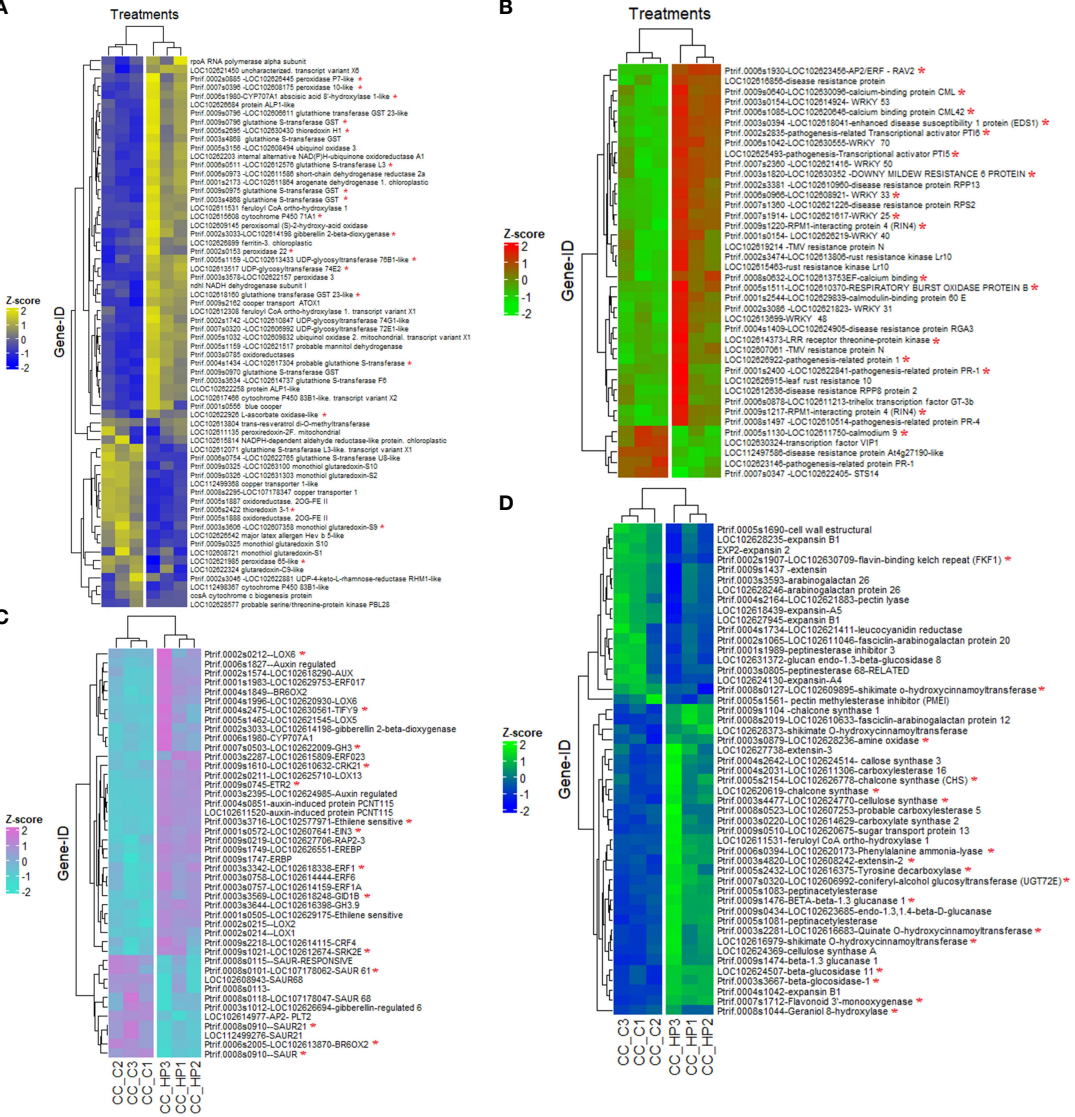
Hierarchical clustering illustrating the expression profiles of the functionally enriched genes associated with highly represented biological events in (Z)-3-HP-exposed CC plants. **(A)** genes related to antioxidant response, **(B)** genes associated with the plant defense response, **(C)** genes related to hormonal signal pathways linked to plant immune defense, and **(D)** genes related to secondary metabolite biosynthesis. The *Z*-score of each gene is shown using a color scale. Gene IDs with *C. sinensis* and *P. trifoliata* official gene symbols are indicated on the right side of each heatmap. In contrast, the bottom side represents the expression profiles in each experimental sample/condition. Genes marked with a red asterisk are part of the gene sets identified in the enriched functional terms GSEA analysis.

Functional analysis reveals a high percentage, approximately 13%, of antioxidant activity in (Z)-3-HP-exposed CC plants (**Figure 5**). The gene set orchestrating this process includes different types of ROS-scavenging enzymes, including ascorbate peroxidases (APXs) such as L-ascorbate peroxidase (*LOC102622926*); glutathione transferases (GSTFs) like *GSTF1* (*LOC102622765*), *GSTF8* (*LOC102617304*), (Ptrif.*0003s4868*), (Ptrif.*0009s0975*), (Ptrif.*0009s0796*), *GSTF23* (*LOC102618160*), and *GST13* (*loc102612576*); thioredoxins such as thiredoxin3 (Ptrif.*006s2422*) and thioredoxin *H1* (*LOC102630430*); glutaredoxins (GRXs) including *GRX10* (*LOC10263100*), *GRX2* (*LOC102631303*) and *GRX9* (*LOC102607358*); and peroxidases (PERs) such as *PER1* (*LOC102617398*), *PER10* (*LOC102608175*), *PER15* (*LOC102613627*), (*LOC102615475*), *PER3*(*LOC102622157*), *PER*-*A2* (*LOC102614803*), *PER7* (*LOC102626445*), *PER 65* (*LOC102621985*), *PER 22* (*Ptrif*.*0002s0153*), most of which exhibit upregulation (**Figures 6**, **7A**). Within the co-expression network, *GRX2*, *GSTF8*, *PER24*, and *PER7* are notably identified as molecular hubs in our biological context (**Supplementary Figure S4**).

### 3.5 Plasma membrane-localized receptor-like kinases (RLKs) in plant-microbe recognition

Plasma membrane-localized receptor-like kinases (RLKs) play indispensable roles in both the recognition of plant-microbe interactions, particularly in the context of PAMP-triggered immunity (PTI), the orchestration of various developmental processes in plants. In the genomic landscape of *P. trifoliata*, a notable assemblage of genes within this family has been delineated, comprising 87 genes encoding leucine-rich repeats (LRRs), 3 genes encoding LRR-RLKs, and 17 genes encoding Cys-rich repeats (CRKs). Conversely, an exploration of the *C. sinensis* genome has revealed the presence of 28 RLKs attributed to the CRKs family, encompassing 18 genes of LRR-RLKs and 10 genes of CRKs. Overall, RLKs represent a 7% enrichment of functional DEGs. Notably, within the functional analysis, RIPK (LOC102614373) and leaf rust resistance proteins RLK5 (Ptrif.0002s2120), involved in the pattern-triggered immunity (PTI) system, were highlighted, emerging in the co-expression network (**Supplementary Figure S4**). Functional and co-expression analysis reveal transcription factors (TFs) and regulatory proteins as key hubs in the plant-pathogen signaling network (**Figures 6**, **7B**, **Supplementary Figure S4**). Key TFs identified in both genomes included pathogenesis-related transcription factors PTI5 (LOC102625493) in *C. sinensis* and PTI6 (Ptrif.002s2835) in *P. trifoliata*, associated with pattern-triggered immunity (PTI), and the TFs GT-3B (LOC102611213) and RAV2 (Ptrif.0006s1930) for basal and specific effector-triggered immunity (ETI). The co-expression network reveals TFs acting as molecular hubs involved in defense responses such as WRKY33 (LOC102608921), WRKY25 (LOC102621617), WRKY31 (LOC102621823), WRKY40 (LOC102626219), WRKY48 (LOC102613699), WRKY50 (LOC102621416), WRKY53 (LOC102614924), and WRKY70 (LOC102630555); and Calmodium plant immune system such as CML30 (LOC102613753), CML42 (LOC102620646), CML8 (LOC102630096), and CML60E (LOC102629839), calcium sensors crucial in triggering defense response (**Supplementary Figure S4**). Moreover, the analysis identifies several major transcriptional regulators highly enriched encoding R proteins, primarily TIR-NB-LRR (NL) family members such as RIN4 (Ptrif.0009s1217), as well as other R proteins such as pathogenesis-related 1 (PR1) (LOC102622841), RPPL proteins, along with regulatory proteins such as EDS1 (enhanced disease susceptibility) (LOC102618041), which regulate basal and effector-triggered immunity (**Supplementary Figure S4**, **Figure 7B**).

### 3.6 Hormone signaling complexity

Nearly 7% of the functional gene sets were associated with hormone signaling (**Figures 6**, **7C**). Among the downregulated hormone pathways, the auxin-responsive genes stand out, such as the *Small Auxin-Up RNA (SAURs)* like *SAUR21* (*Ptrif.0008s0910*), *SAUR* (*Ptrif.0008s0910*), *SAUR68* (*LOC107178047*), and *SAUR61* (*LOC107178062*), along with the APETALA transcription factor *AP2-PLT2* (*LOC102614977*). However, a notable overrepresentation of specific auxin-induced genes like LOC102624985 and Ptrif exists*.0004s0851*, *LOC102611520*, and *GH3* (*LOC102622009*), which catalyzes the synthesis of IAA. Our investigation into ethylene-responsive genes uncovered significant upregulation of a gene set comprising 11 and 24 functionally enriched DEGs in the *C. sinensis* and *P. trifoliata* genomes, respectively, indicating their regulatory significance. Notably, key components such as ethylene response transcription factors (*ERF017* (*LOC102629753*), *ERF1A* (*LOC102614159*), *ERF023* (*LOC102615809*), *ETR2* (*Ptrif.0009s0745*), *ERF1* (*LOC102618338*), *ERF6* (*LOC102614444*)) and *ethylene insensitive 3 (EIN3)* (*LOC102607641*) were identified within this gene set, exerting regulatory functions in the co-expression network (**Supplementary Figure S4**). These genes are crucial for plant responses to various stresses, particularly in triggering and modulating plant immune responses. The analysis of the jasmonate signaling pathway identified the upregulation of *TIFY9* (*LOC102630561*) in both genomes, a member of the JAZ-like protein family, as a crucial hub in the JA signaling pathway and a central node in the co-expression network. Additionally, key members of the *lipoxygenase (LOX)* gene family, including *LOX6* (*LOC102620930*), *LOX5* (*LOC102621545*), *LOX13* (*LOC102625710*), *LOX2* (*Ptrif.0002s0215*), and *LOX1* (*Ptrif.0002s0214*), were found to be functionally enriched and centrally regulated within the co-expression network (**Figures 6**, **7C**, **Supplementary Figure S4**). In the brassinosteroid-related DEGs, *BR6OX2* (*Ptrif.00062005*) was downregulated and functionally enriched in the *P. trifoliata* background. Additionally, three gibberellin-related genes, including *gibberellin regulated-6* (*LOC102626694*) downregulated, as well as *GA2OX2* (*LOC102614198*) and *GIB1* (*LOC102618248*) (the gibberellin receptor) functionally enriched and upregulated. The study revealed four salicylic acid (SA)-related genes upregulated, with *PAL* (*LOC102620173*) highlighted as a key player in SA biosynthesis and a hub in the co-expression network. Additionally, 14 genes encoding *Salicylic acid carboxyl methyltransferases*, all upregulated, were found to be functionally relevant in SA-related processes (**Figure 6**). Functional enrichment of upregulated *SRK2E (LOC102612674)* gene, encoding serine/threonine kinases, suggests that there is an activation in the abscisic acid (ABA) signaling pathway. These kinases serve as central nodes activated by ABA during stress, initiating the phosphorylation of downstream proteins and the stress response cascade. Additionally, the upregulated and functionally enriched enzyme *CYP707A3* and *CYP707A1* (*Ptrif.0006s1980*), involved in ABA biosynthesis, was identified in the co-expression network (**Supplementary Figure S4**).

### 3.7 Enzymes regulating secondary metabolite biosynthesis and plant development

The gene set under discussion is essential for the biosynthesis of various secondary metabolites, including flavonoids, isoflavonoids, phenylpropanoids, amines, polyamines, terpenoids, and specialized compounds like glycosides, tannins, saponins, resins, and phenolic compounds. Enzymes within this set, notably *chalcone synthase* (*CHS*) (*LOC102626778*), *flavonoid monooxygenase* (*Ptrif.0007s1712*), *tyrosine decarboxylase* (*LOC102616375*), and *phenylalanine ammonia-lyase* (*PAL*) (*LOC102620173*), play central roles and are upregulated. In addition, *geraniol-8-hydroxylase* (*Ptrif.0008s1044*), a cytochrome P450 enzyme critical for terpenoid biosynthesis and wound response regulation, is upregulated and functionally enriched (**Figures 6**, **7D**).

Plant developmental processes were enriched through the upregulation of carboxylate synthesis proteins for sugar synthesis during photosynthesis and the sugar transport proteins like *STP13* (*LOC102620675*). Genes associated with cell wall synthesis, including *cellulose synthase A* (*CSLG3*) (*LOC102624770*), *expansin-B1* (*Ptrif.0004s1042*), *extensin-2* (*LOC102608242*), *extensin-3* (*LOC102627738*), and *pectin acetylesterases* (*Ptrif.0005s1083*), were functionally upregulated. Notably, the *Scarecrow-like protein SCL29* (*LOC102626640*), involved in plant development, was upregulated, in contrast to the downregulation of *Arabinogalactan proteins* (*AGP*) like *AGP26* (*LOC102628246*), (*Ptrif.0003s3593*), which regulate cell wall flexibility and elasticity (**Figures 6**, **7D**).

Our findings revealed a regulatory mechanism governing callose accumulation in CC plants induced by *(Z)-3-HP*, involving five upregulated and functionally enriched genes implicated in callose degradation. These genes include *beta-glucosidase-1* (*Ptrif.0003s3667*), *beta-glucosidase-11* (*LOC102624507*), *1.4-beta-D-glucanase* (*Ptrif.0009s0434*), and *beta-1.3-glucanase* (*Ptrif.0009s1474*), (*Ptrif.0009s1476*), all identified within the *P. trifoliata* background and *beta-glucosidase-11* (*LOC102624507*) identify in *C. sinensis* genome. Additionally, one gene associated with callose degradation, *glucan endo 1.3 beta-glucosidase 8* (*LOC102631372*), showed downregulation in the *C. sinensis* genome. In contrast, two genes encoding callose such as *callose synthase A* (*LOC102624369*) and *callose synthase-3* (*LOC102624514*), were upregulated and functionally enriched within the *C. sinensis* genome (**Figure 7D**).

The functional enrichment downregulated in the KEGG pathway “photosynthesis” in the *C.sinensis* genome is remarkable. The genes that make up this pathway are cytochrome b6/f complex subunit VIII (*petN*), oxygen-evolving enhancer protein (*LOC102621051*), and photosystem II protein D1(*psbA*).

### 3.8 Callose deposition intensity and β-1,3-glucanase activity

**Figure 8:**
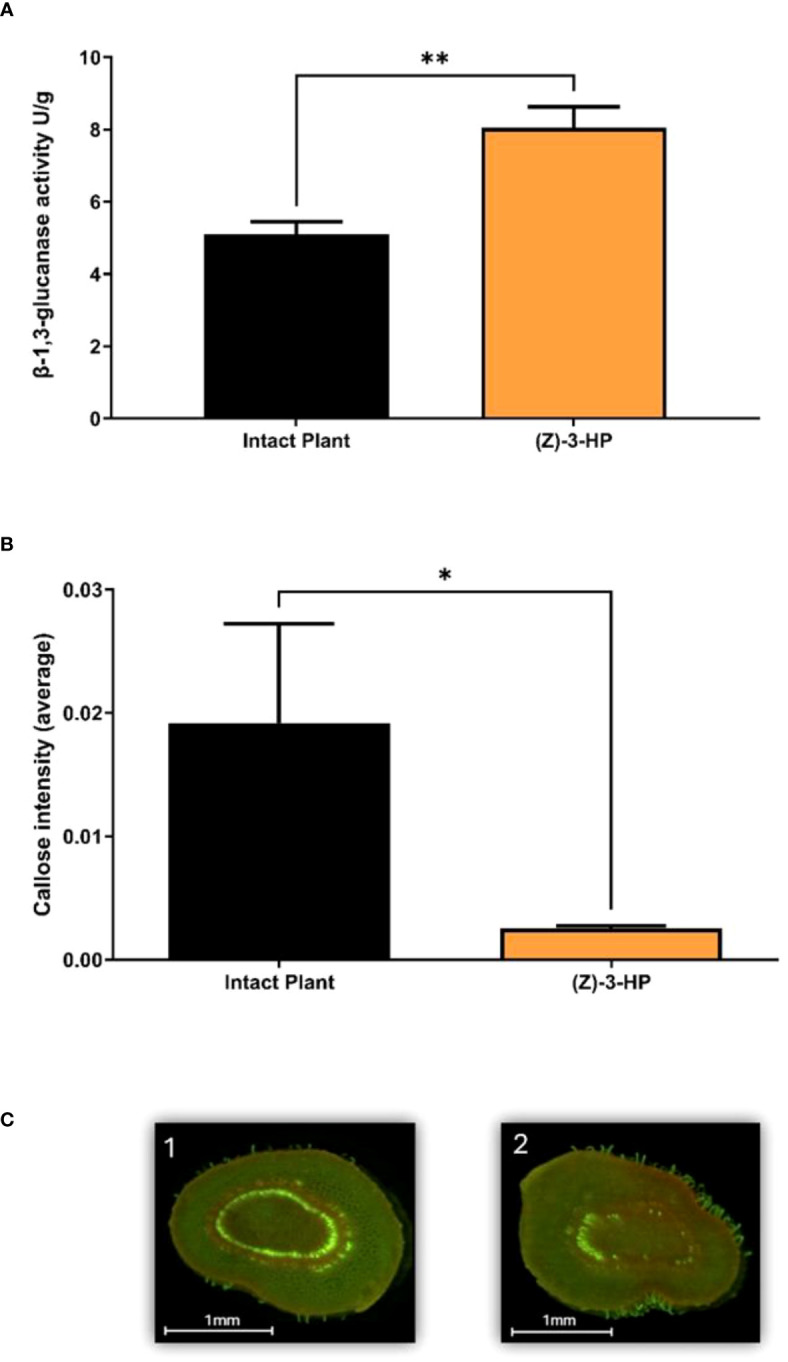
Callose Deposition Intensity and β-1,3-Glucanase activity. **(A)** Quantification of β-1,3-glucanase enzyme activity assessed using the DNS (3,5-dinitrosalicylic acid) method in a microplate assay, which measures reducing sugars released from Laminarin (*t*-test; *P* < 0.05) the asterisk (*) represents a Pvalue<0.05, **(B)** Callose intensity was quantified corresponding to stained callose cross sections of CC plants and analyzed for the number of pixels using GIMP (GNU Image Manipulation Program) (*t*-test; *P* < 0.05) the asterisks (**) represents a Pvalue<0.01. **(C)** Stained cross sections of CC stems showing callose deposits were observed under the epifluorescence microscope with a UV filter under the following conditions: 1) intact plants, 2) (Z)-3-HP-exposed plants.

Exposure of CC plants to (Z)-3-HP volatile caused a significant increase in the activity of the β-1,3-glucanase enzyme compared to intact plants without volatile exposure (*t* = 3.843; df = 1, 8; *P* = 0.0049) (**Figure 8A**). Consequently, by examining the intensity of callose deposition through aniline blue staining and epifluorescence analysis and quantifying the fluorescent areas of callose deposits in the stem sections, intact plants exhibited more callose deposits throughout the vascular bundle in the stem of the apical part of the plant (**Figures 8B, C**). In contrast, (Z)-3-HP-exposed plants showed a significant decrease in callose deposits in the vascular bundle (*t* = 2.828; df = 1, 6; *P* = 0.030) (**Figures 8B, C**).

### 3.9 Plant selection by pests and natural enemies

**Figure 9:**
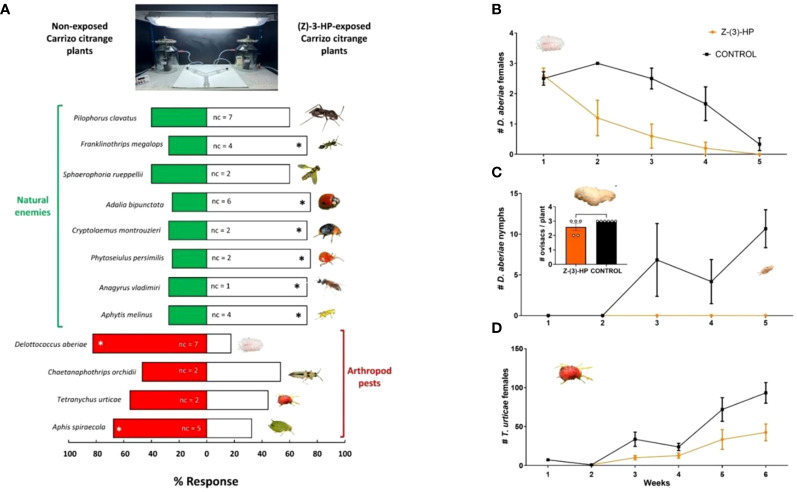
Behavioral response of citrus arthropod pests and natural enemies and effects on *D. aberiae* and *T. urticae* to intact or (Z)-3-HP-exposed CC plants. **(A)** Response of citrus arthropod pests (*Delottococcus aberiae*, *Chaetanaphothrips orchidii*, *Tetranychus urticae*, and *Aphis spiraecola*) and natural enemies (*Aphytis melinus*, *Anagyrus vladimiri*, *Phytoseiulus persimilis*, *Cryptolaemus montrouzieri*, *Adalia bipunctata*, *Sphaerophoria rueppellii*, *Franklinothrips megalops*, and *Pilophorus clavatus* to intact or (Z)-3-HP-exposed CC plants in a Y-tube olfactometer. Asterisks indicate significant differences in the distribution of side-arm choices (*χ* ^2^ tests; *P* < 0.05). “nc” indicates the number of tested females that did not make a choice. **(B)** Number of *D. aberiae* females (± SE), **(C)** number of *D. aberiae* nymphs (± SE), and **(D)** number of *T. urticae* females (± SE) for (Z)-3-HP-exposed CC plants and control CC plants (GLMM; *P* < 0.05). **(B)** depicts the number of ovisacs formed per plant in each treatment (*t*-test; *P* < 0.05).

Among the 12 arthropod species tested, 8 altered their plant selection behavior due to prior exposure to (Z)-3-HP (**Figure 9A**). Two of the tested pests, *Delottococcus aberiae* and *Aphis spiraecola*, showed repellence towards CC plants previously exposed to (Z)-3-HP and significantly preferred choosing the control plants without exposure (*χ* ^2^ = 16.90, *P* < 0.0001; *χ* ^2^ = 4.90, *P* = 0.027, respectively *Chaetanaphothrips orchidii* and *Tetranychus urticae* did not show a preference for either treatment (*χ* ^2^ = 0.200, *P* = 0.655; *χ* ^2^ = 0.556, *P* = 0.456, respectively). Among the natural enemies, 6 species were attracted to plants previously exposed to (Z)-3-HP compared to the control. These species were *Franklinothrips megalops* (*χ* ^2^ = 8.10; *P* = 0.004), *Adalia bipunctata* (*χ* ^2^ = 10.00; *P* = 0.002), *Cryptolaemus montrouzieri* (*χ* ^2^ = 8.10; *P* = 0.004), *Phytoseiulus persimilis* (*χ* ^2^ = 10.00; *P* = 0.002), *Anagyrus vladimiri* (*χ* ^2^ = 8.10; *P* = 0.0044) and *Aphytis melinus* (*χ* ^2^ = 8.10; *P* = 0.004). Only two of the natural enemies, *Pilophorus clavatus* (*χ* ^2^ = 2.20, *P* = 0.138) and *Sphaerophoria rueppellii* (*χ* ^2^ = 1.60, *P* = 0.206), did not show a preference for either plant treatment.

### 3.10 Citrus pests performance

The number of *D. aberiae* females released on plants subjected to both treatments exhibited a significant decrease in the (Z)-3-HP-exposed group compared to the control treatment (*F* = 9.961, df = 1, 53, *P* = 0.003) (**Figure 9B**). However, no significant differences were observed in the number of ovisacs formed by these females between the two treatments (*t*= 1.809, df =1, 9; *P*= 0.1039), as almost each female formed its corresponding ovisac (**Figure 9C**). Notably, in the control treatment, the ovisacs resulted in the progressive hatching of *D. aberiae* nymphs over time. In contrast, in the (Z)-3-HP-exposed CC plants, no hatching of nymphs from these ovisacs was recorded (**Figure 9C**). Regarding the two-spotted spider mite T. urticae, mite populations increased more rapidly in the control treatment than in the (Z)-3-HP-exposed CC plants treatment (*F* = 79.994, df = 1, 70, *P* < 0.0001) (**Figure 9D**).

## 4 Discussion

**Figure 10:**
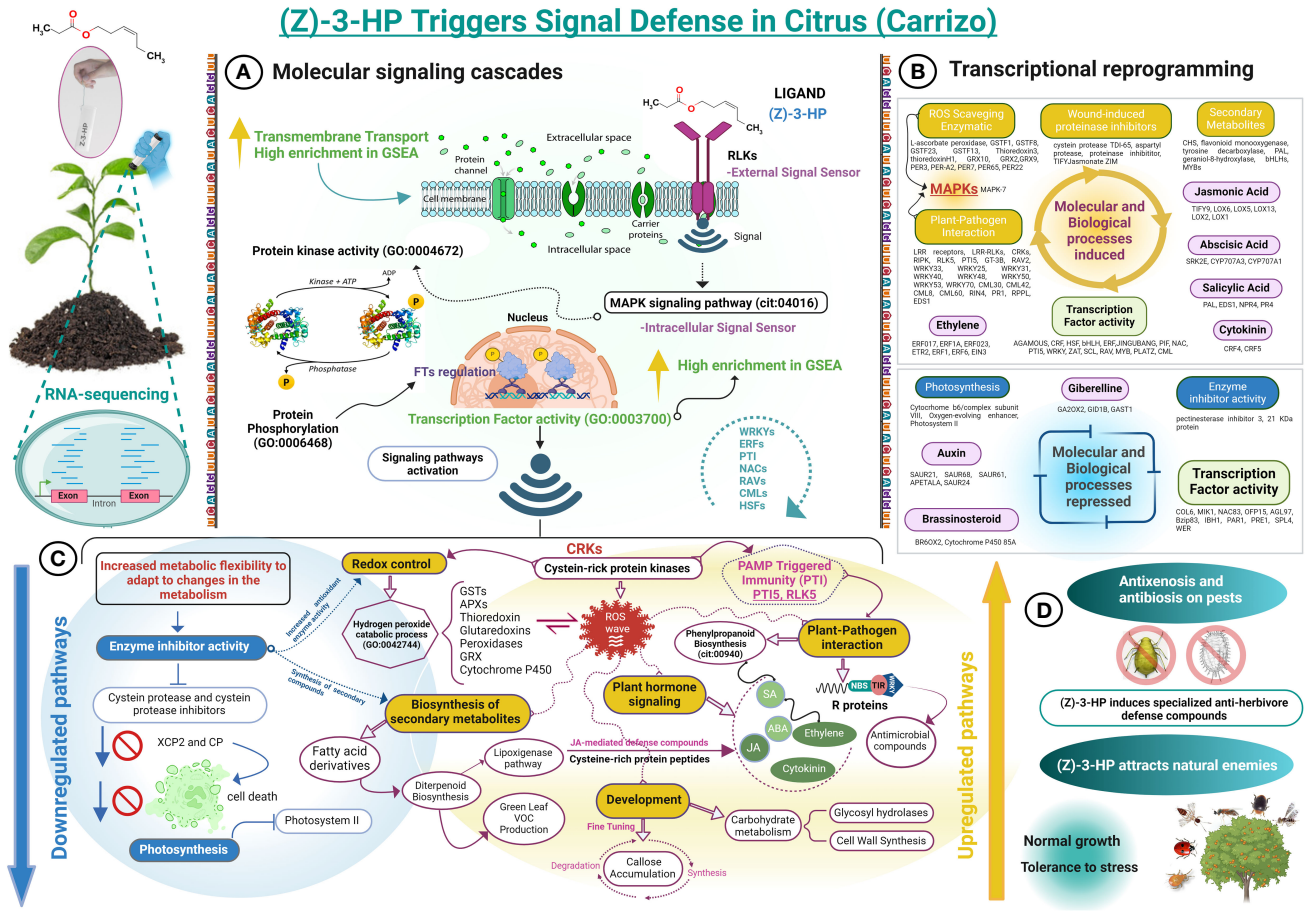
Flowchart of the regulation of defense response in (Z)-3-HP-exposed CC plants. **(A)** Exposure to (Z)-3-HP compound activates receptor-like kinases, which detect and transmit extracellular signals via protein phosphorylation. These proteins act as second messengers, amplifying the signal through diverse intracellular signaling pathways. This signaling cascade modulates the expression of transcription factors and regulatory genes, thereby adjusting the cellular response. **(B)** Differentially expressed genes, both upregulated and downregulated, that are functionally enriched in various molecular and biological processes. **(C)** Downregulation of cysteine proteases and inhibitors modulates the expression of defense-related genes and inhibits programmed cell death. Conversely, upregulation of cysteine-rich receptor-like kinases (CRKs) functions as an early warning system, detecting stressors and activating immune-related signaling pathways. The regulation of callose accumulation, with a predominance of genes upregulated genes for its degradation over its synthesis, ensures efficient nutrient transport during an activated defense response. In addition, the upregulation of glycosyl hydrolases enhances starch degradation, providing an energy source. **(D)** Exposure to (Z)-3-HP compound alters arthropod behavior in citrus plants, demonstrating antibiosis and antixenosis effects on pests such as *Delottococcus aberiae* and *Aphis spiraecola*, while attracting natural enemies, including *Franklinothrips megalops*, *Adalia bipunctata*, *Cryptolaemus montrouzieri, Phytoseiulus persimilis, Anagyrus vladimiri*, and *Aphytis melinus*.

This study reveals that exposure to (Z)-3-HP in CC triggers a cascade of significant defensive responses (**Figure 10**), highlighting the importance of this compound in conferring resistance against both biotic and abiotic stressors. These findings align with prior research that underscores the pivotal role of VOCs in inter- and intraspecific communication, promoting the attraction of natural enemies of herbivores and bolstering plant defenses (López-Gresa et al., 2018; Turlings and Erb, 2018; Pérez-Hedo et al., 2021; Riahi et al., 2022; Payá et al., 2024). In this study, we have demonstrated how exposure to (Z)-3-HP enhances plant defenses against two major citrus pests: the spider mite, *T. urticae*, and the South African citrus mealybug, *D. aberiae*. The contribution of *P. trifoliata* to the gene expression profile of the CC hybrid in response to the (Z)-3-HP stimulus underscores the distinct value of this parental species in activating defense mechanisms against both biotic and abiotic stresses. *Poncirus trifoliata* has been shown to confer superior resistance to various citrus pests, such as the citrus leafminer *Phyllocnistis citrella* Stainton (Lepidoptera: Gracillariidae) (Jacas et al., 1997; Santos et al., 2019) and the Asian citrus psyllid *Diaphorina citri* Kuwayama (Hemiptera: Liviidae) (Hall et al., 2017; Urbaneja-Bernat et al., 2020). Additionally, *P. trifoliata* exhibits greater tolerance to devastating diseases like huanglongbing (HLB) than other citrus rootstocks (Rawat et al., 2017; Hall et al., 2019; Curtolo et al., 2020). Despite *P. trifoliata*’s resistance to various pests and diseases, CC has been identified as a rootstock susceptible to several pests and pathogens (Bruessow et al., 2010; Urbaneja-Bernat et al., 2020). Therefore, implementing strategies that activate the innate defenses of *P. trifoliata* could be crucial for enhancing resistance to CC. This is particularly relevant in Spanish citrus cultivation, where approximately 61% of plantations utilize CC as a rootstock (Tallón-Vila, 2015). An important future objective would be to investigate how exposure to volatiles, such as (Z)-3-HP, modulates defenses in grafted plants and determine the role played by the rootstock in activating these defensive responses.

Plant defense against pests involves a complex signaling network that triggers specific physiological and molecular responses. The jasmonate (JA) signaling pathway is pivotal in activating defenses against herbivores (Glazebrook, 2005; Bari and Jones, 2009). A prominent example in our results is a clear shift towards the upregulation of secondary metabolite biosynthesis, emphasizing the synthesis of fatty acid-derived compounds, activation of lipoxygenase pathway, and accumulation of specialized anti-herbivore defense compounds. These findings are consistent with our previous study on the transcriptome of (Z)-3-HP-exposed tomato plants (Pérez-Hedo et al., 2021), which revealed the activation of the Lipoxygenase (LOX) pathway associated with antiherbivore defense in tomato (Feussner and Wasternack, 2002; Bari and Jones, 2009; Şahin-Çevik et al., 2014; Agut et al., 2015). Additionally, genes of the WRKY transcription factor family, significantly regulated in our research, are known for their role in mediating the response to JA and regulating defense against herbivores and pathogens (Zheng et al., 2006; Birkenbihl et al., 2012). For instance, the interplay between WRKY33 and JA signaling genes is crucial in orchestrating plant defense mechanisms. WRKY33 serves as a central regulator by controlling the expression of LOX enzymes (Birkenbihl et al., 2012). In our study, upregulated WRKY33 (LOC102608921) physically interacts with *LOX1* (Ptrif.0002s0214), *LOX2* (Ptrif.0002s0215), and LOX6 (LOC102620930) (**Supplementary Figure S4**), acting as a master regulator over these crucial *LOX* components. On the other hand, another prominent WRKY in our transcriptome was WRKY70 (Ptrif.0006s1042), which was upregulated in the *P. trifoliata* genome and was identified by Peng et al. (2021) as the primary TF gene that plays a vital role in *P. trifoliata* tolerance to HLB.

Another relevant pathway in our research is ethylene signaling, characterized by a significant overrepresentation of Ethylene Response Factors (EFRFs), which are involved in modulating biotic and abiotic stress responses (Wang et al., 2002; Bari and Jones, 2009). The crosstalk between JA and ethylene pathways, both pivotal for plant defense, underscores the intricate nature of plant signaling networks and their capacity to finely tune defensive responses depending on the encountered stressors (Fujita et al., 2006). ERFs contribute to plant defense against herbivores and play a crucial role in defense against pathogens by regulating the expression of genes encoding pathogenesis-related (PR) proteins, which directly combat pathogens or fortify cell structures to impede their progress. This regulation occurs through crosstalk with the salicylic acid (SA) pathway, a well-known essential pathway in plant defense against biotrophic pathogens. In our study, we noted the upregulation of genes related to SA biosynthesis and signaling, alongside a notable increase in the expression of genes associated with immune system activation, particularly Leucine-Rich Repeat Receptor-Like Kinases (LRR-RLKs) and Cysteine (Cys)-rich repeat (CRKs), which are prevalent in the *P. trifoliata* background. Similar findings were reported in HLB-infected lime [*Citrus australasica* F.Muell. (Sapindales: Rutaceae)] (known to be tolerant to HLB) by Weber et al. (2022). These proteins are pivotal in regulating basal immunity and systemic acquired resistance (SAR), constituting a long-lasting defense response induced by SA signaling.

A notable aspect is the identification of genes associated with the mitogen-activated protein kinase (MAPK) signaling pathway, which is involved in signal transduction following pathogen detection (Meng and Zhang, 2013). Activation of this pathway results in the expression of defensive genes. It is closely intertwined with reactive oxygen species (ROS) production, serving as both cellular signaling and a direct defense against invaders (Kovtun et al., 2000). As evidenced in our study, the activation of antioxidant pathways and the upregulation of genes related to defense against herbivores imply a shared underlying mechanism in managing oxidative stress in plants.

In plant defensive responses to biotic and abiotic stressors, polysaccharides such as galactose and callose play crucial roles in modulating resistance and cellular signaling (Cervilla et al., 2007). In our study, we observed the regulation of genes related to the biosynthesis and degradation of these polysaccharides. Genes associated with the galactose metabolic pathway exhibited differential regulation, suggesting potential involvement in cell wall reconfiguration and the activation of defense responses. The regulation of the genes related to callose synthesis and degradation, observed in our samples exposed to (Z)-3-HP, underscores its importance in CC’s defensive response. Specifically, the upregulation of genes involved in callose synthesis suggests a mechanism for cell wall reinforcement as a direct response to volatile stimuli. Conversely, the regulation of genes associated with callose degradation could reflect the fine-tuning of this reinforcement in response to different phases of biotic stress. This balance between polysaccharide synthesis and degradation, such as galactose and callose, underscores plants’ complex and dynamic strategy to maintain structural integrity and defense signaling against environmental challenges.

The activation and function of antioxidants in plants, especially under stress conditions, have become an increasingly important focus due to their essential ability to mitigate cellular damage caused by reactive oxygen species (ROS) (Lamb and Dixon, 1997; Blokhina et al., 2003). In our research with CC exposed to (Z)-3-HP, we detected significant positive regulation of genes linked to antioxidant response, including those encoding ROS scavenging enzymes such as ascorbate peroxidase (APX), superoxide dismutase (SOD), glutathione reductases (GRs), peroxidases, and thioredoxin glutaredoxins. These enzymes play a fundamental role in plant redox homeostasis (Gill & Tuteja, 2010), suggesting that exposure to certain volatile compounds can enhance the plant’s detoxification antioxidant system, preparing them to cope more effectively with oxidative stress derived from pathogen attacks and unfavorable environmental conditions (Wei et al., 2019). This system protects plant cells from oxidative damage and intervenes in cellular signaling, regulating broader defensive responses, indicating that inducing a heightened antioxidant state may be a valuable defense strategy for developing more resilient crops capable of withstanding various biotic and abiotic stressors. This interest becomes more pronounced in diseases like HLB, where managing oxidative stress induced by pathogens emerges as a pivotal strategy to alleviate disease symptoms (Hijaz et al., 2020; Ma et al., 2022). Infection by *Candidatus* Liberibacter asiaticus (*C*Las), the causative agent of HLB, triggers a systemic and persistent immune response in phloem tissue, involving callose deposition, generation of ROS such as H_2_O_2_, and activation of genes associated with immunity. These findings suggest that HLB can be viewed as an immunity-mediated disease susceptible to mitigation by applying antioxidant compounds. This underscores the role of antioxidants as direct protectors against oxidative damage and as modulators of plant immune responses to pathogens Ma et al., (2022). Nehela & Killiny (2023) demonstrated that γ-aminobutyric acid (GABA) accumulation in *C. sinensis* significantly contributed to the plant’s response against *C*las, modulating multiple metabolic pathways and optimizing the redox state through enzymatic and non-enzymatic antioxidant defenses. This defense strategy effectively neutralizes the harmful effects of ROS induced by *C*Las infection. Such findings suggest that modulating plant immune responses through oxidative stress management offers a promising approach for HLB control, underscoring the importance of further exploring antioxidants for plant health and disease resistance. Thus, interest in exploring the role of volatile compounds in enhancing citrus antioxidants becomes increasingly relevant.

In summary, our work provides a promising outlook for developing pest management strategies centered around exposure to natural plant volatiles, offering a sustainable alternative to chemical pesticides. However, there are still some questions and challenges to be addressed for large-scale implementation: i) The effectiveness of VOCs can vary depending on environmental conditions, crop type, and pest species, ii) Implementing VOC dispensers on a large scale can be costly and logistically challenging, although current technology should mitigate this issue, iii) The rapid decomposition and climate-influenced dispersion of VOCs require further investigation in open fields iv) While VOCs could affect non-target organisms such as pollinators, our preliminary results are promising, but large-scale observations are necessary and v) Additionally, regulatory and acceptance barriers from farmers could hinder the adoption of these new technologies. However, engaging with regulatory bodies to establish clear guidelines and conducting outreach programs to educate farmers about the benefits and usage of VOCs can facilitate smoother adoption. Finally, this study initiates new pathways for research in plant genomics and genetic enhancement, underscoring the necessity to delve deeper into the role of volatiles in plant defense and their potential optimization for cultivating resilient crops.

## Data availability statement

The datasets presented in this study can be found in online repositories. The names of the repository/repositories and accession number(s) can be found below: https://www.ncbi.nlm.nih.gov/, PRJNA1092419.

## Author contributions

MP-H: Conceptualization, Formal analysis, Funding acquisition, Investigation, Methodology, Project administration, Resources, Supervision, Writing – original draft, Writing – review & editing. CG-G: Validation, Formal analysis, Investigation, Methodology, Software, Writing – original draft, Writing – review & editing. MF-G: Investigation, Methodology, Writing – review & editing. RO-F: Investigation, Methodology, Writing – original draft, Writing – review & editing. AU: Conceptualization, Funding acquisition, Investigation, Methodology, Project administration, Resources, Supervision, Writing – original draft, Writing – review & editing.
