# Supplementary material for: Plant volatile-triggered defense in citrus against biotic stressors
Source: Front Plant Sci. 2024 Jul 10;15:1425364. doi: 10.3389/fpls.2024.1425364 (PMC11266131; doi:10.3389/fpls.2024.1425364)
Supplement: Supplementary file 1 [file DataSheet_1.docx]

Plant volatile-triggered defence in citrus against biotic stressors

Meritxell Pérez-Hedo^1*^, Carolina Gallego-Giraldo^1^, María Ángeles Forner-Giner^2^, Raúl Ortells-Fabra^1^, Alberto Urbaneja^1^

^1^Instituto Valenciano de Investigaciones Agrarias (IVIA). Centro de Protección Vegetal y Biotecnología, CV-315, Km 10.7, 46113 Moncada, Valencia, Spain

^2^Instituto Valenciano de Investigaciones Agrarias (IVIA), Centro de Citricultura y Producción Vegetal, CV-315, Km 10.7, 46113 Moncada, Valencia, Spain

*Corresponding autor: perez_merhed@gva.es

Supplementary Material

# Supplementary Methods S1 – Plants and arthropods

The Carrizo citrange (CC) material was obtained from seeds and cultivated in plastic pots measuring 8 x 8 x 8 cm. The substrate comprised 70% black peat and 30% perlite. The plants were nurtured for three months until they attained a height of approximately 30 cm, displaying the expansion of eight to nine leaves, indicative of a specific developmental stage suitable for subsequent bioassays. Cultivation occurred in a controlled environment maintained at 25 ± 1ºC, relative humidity of 60%, and a photoperiod of 14:10 h (L:D).

Pest specimens were obtained from colonies maintained at the Instituto Valenciano de Investigaciones Agrarias (IVIA) facilities. *Delottococcus aberiae,* *C. orchidii,* and *T. urticae* were reared on lemon fruits, whereas *A. spiraecola* was reared on mandarine clementine cv. Clemenules grafted on CC of 2-year-old trees. All colonies were housed in climatic chambers at 25 ± 2 ºC, 60-80% RH, and a 14:10 h (L:D) photoperiod. *Aphytis melinus*, *A. vladimiri*, *P. persimilis*, *C. montrouzieri*, *S. rueppellii* and *A. bipunctata* were provided by Koppert Biological Systems (Aguilas, Spain). The predators *F. megalops* and *P. clavatus* were sourced from established colonies at IVIA. These colonies used *Ephestia kuehniella* Zeller eggs (Lepidoptera: Pyralidae) as alternative prey.

# Supplementary Methods S2 – RNA-seq data processing, functional annotation and analysis

## RNA-Seq dataset processing and differential expression analysis.

The raw sequencing data underwent quality filtering using FastQC v0.11.9 (<https://www.bioinformatics.babraham.ac.uk/projects/fastqc/>) to retain reads with a Phred Score ≥20. Trimmomatic v0.38^54^ was then utilised to trim adapter sequences, clip lower-quality 3´-ends, and mask low-complexity or low-quality reads using a sliding window approach. Trimming parameters included a minimum length of 100bp, a sliding window size of 4, and an average quality score of less than 15. Reads failing these criteria were excluded, resulting in a trimmed dataset suitable for subsequent analysis. The high-quality clean reads were mapped and aligned to the reference genomes of *Citrus Sinensis* Csi_valencia_1.0 and *Poncirus Trifoliata* Ptrifoliata_565_v13 using HISAT2 v2.1.0 with Bowtie2 aligner v2.3.4.1^55^. The known genes and transcripts were assembled using StringTie v1.3.4b^56^. During the review process of the article, a new genome of *C. sinensis* appeared that does not invalidate the biological data obtained with the genomic tool we used. It would be interesting if future studies reference this new genome (DVS_A1.0 genome in NBCI), as it is more advanced than the one used in this work.

Gene/transcript abundance was determined based on mapped read counts, excluding intronic regions, and normalized to Fragment Per Kilobase of Transcript per Million Mapped Reads (FPKM) and Transcripts Per Kilobase Million (TPM). Genes were considered expressed if both FPKM and TPM values were greater than zero. FPKM and TPM values for filtered transcripts were log-transformed using log2 with an offset of 1, and calculation and normalisation were performed using featureCounts v.2.0.6 software with the read count list extracted from the output file for each reference genome.

To reduce systematic bias in the analysis, size factors were estimated from the read count data of each reference genome using the calcNormFactors method. The read count data were then normalized for each genome using the Trimmed mean of M-values (TMM) method, implemented in the edgeR library (v.4.4.0). After normalization, statistical analysis was performed using the TMM-normalized counts adopted for exact texts (exactTest) provided by edgeR for the comparison pairs. The Benjamini and Hochberg method was applied for p-value adjustment to calculate the false discovery rate (FDR). A gene was considered significantly differentially expressed if the absolute value of the fold change was │log_2_│≥ 2, with an FDR<0.05 and an exactTest raw *p*-value of <0.05.

Correlation matrix, multidimensional scaling (MDS) and hierarchical clustering analyses were performed using the preprocessCore R library to assess the biological reproducibility of the sample replicates.

## Functional analysis: Gene Ontology, GSEA, and gene expression profiles.

The functional annotation of differentially expressed genes (DEGs) was analyzed using the DAVID tool (<http://david.abcc.ncifcrf.gov/>). Parameters for accurate Gene Ontology (GO) term assignment included the utilization of Fisher's exact test with Benjamini-Yekutieli adjustment^57^ for false discovery rate (FDR) control (*alpha* = 0.05) and a minimum mapping threshold of 5 for gene support. Statistically significant GO terms from DAVID were then processed using the REVIGO tool ([http://revigo.irb.hr](http://revigo.irb.hr/)) to reduce redundancy. Gene functions and pathways were annotated using the Kyoto Encyclopedia of Genes and Genomes (KEGG) Pathway Database (<http://genome.jp/kegg/pathway.html>).

Gene set enrichment analysis (GSEA) was conducted using the R Bioconductor package GAGE (Generally Applicable Gene-set Enrichment Analysis) v.3.17 to analyze enriched gene sets and pathways. For this analysis, previously normalized gene expression data and fold change values were used, both of which were derived from differential gene expression (DEG) analysis performed with the edgeR package, as described previously. Visualization and exploration of enriched gene sets were carried out using the ClueGO application in Cytoscape v.3.7.1. A two-tailed hypergeometric test with Bonferroni step-down correction and a kappa score threshold of 0.4 were employed to highlight functional term connectivity.

A co-expression network analysis was performed to identify key genes with coordinated expression patterns across the sample set. Orthologs of *C. sinensis* and *P. trifoliata* genes related to *Arabidopsis* genes were predicted using BlastP with an e-value cutoff of less than 1e-20. DEGs obtained from functional enrichment analysis were utilised for ortholog prediction. The *C. sinensis* and *P. trifoliata* orthologs were then mapped to the *Arabidopsis* protein-protein interaction (PPI) network using the STRING v11.5 database ([http://string-db.org](http://string-db.org/)) to identify and visualise the co-expression network. Expression patterns and clusters among genes involved in key biological events were visualised using the ComplexHeatmap v2.16.0 and ggplot2 v3.1.1 packages in R.

## Validation of DEGs by real-time quantitative PCR.

To validate the RNA-seq data, a subset of ten up-regulated and ten down-regulated genes was randomly selected for validation using qRT-PCR. The same DNase-treated RNA samples utilised for library preparation and RNA sequencing were employed for the qPCR reaction. Two technical replicates were executed for each sample. The *GAPDH* and *EF1* genes were selected as internal standards. Forward and reverse primer sequences were devised using OLIGO Primer Analysis Software (DBA Oligo, Inc., CO, United States) and are detailed in Table S1. Real-time PCR was conducted on the LightCycler 480® system (Roche Molecular Systems, Inc., Switzerland) with the following experimental run protocol: (95ºC for 10 minutes), 40 cycles (95ºC for 15 seconds, 60ºC for 10 seconds, 72ºC for 60 seconds), (ramping from 60ºC to 95ºC at a heating rate of 0.1ºC per second), and 40ºC for final annealing. The relative expression levels of the selected 20 genes were normalised to the expression levels of *GADPH* and *EF1* genes using the 2-ΔΔCt method. Pearson correlation analysis was conducted using the [log2] fold change (FC) values obtained from RNA-seq and qPCR for each selected gene.

# Supplementary Methods S3 – Y-tube olfactometer

The Y-shaped olfactometer (manufactured by Analytical Research Systems, Gainesville, FL) consisted of a glass tube with an inner diameter of 2.4 cm and a base length of 14.5 cm, with each arm of the Y extending 11 cm. The arthropod under examination was positioned at the base of this Y-shaped tube, which connected to two 5-litre glass jars via corrugated plastic tubing with an outer diameter of 0.8 cm. CC plants were randomly assigned to these jars: one contained (Z)-3-HP-previously exposed plants (as described above), while the other housed non-exposed control plants. Following the placement of the CC plants, both jars were hermetically sealed and connected to a unidirectional air pump. This pump introduced humidified air, purified through water at a consistent pressure of 8 bar, produced by an electric compressor. This setup facilitated an airflow of 160 l/min. The confluence point in the Y-shaped tube intersected the unidirectional airflow from each jar, permitting the insect to sample the air directly. The olfactometer chamber was maintained in darkness, with illumination provided by four 60 cm fluorescent tubes (L18W / 765; OSRAM, Berlin, Germany) positioned 40 cm above the tube.

After recording five responses, the Y-tube was rinsed with soapy water, followed by acetone, and left to dry for 5 minutes. The odour sources were subsequently switched between the left and right-side arms to minimise any spatial effects on choice. The two types of plants (intact and exposed) were used only once to test the response of 10 females and then were replaced with new plants. The Y-tube experiment was conducted under the following environmental conditions: 23 ±2 °C and 60 ± 10% relative humidity.

# Supplementary Figure and table captions

**Supplementary Table S1.** List of genes selected to validate the RNA-seq data by qPCR, the list of the oligos sequence of each gene and its amplicon length are indicated.

**Supplementary** **Figure S1.** **Data quality check.** Raw data includes read counts and FPKM normalized reads from control plants and (Z)-3-HP-exposed plants. After excluding 2,098 *C. sinensis* and 2,045 *P. trifoliata* genes with reading counts equal to or less than zero, 16,846 *C. sinensis* and 18,525 *P. trifoliata* genes remained for analysis. The number of genes passing the filter is highlighted in blue, while the number of genes filtered in each sample condition is shown in red.

**Supplementary** **Figure S2.** **(a)** Correlation matrix was used to measure the similarity between samples under two experimental conditions (CC_C: control plants and CC_HP: (Z)-3-HP). This matrix was constructed using Pearson’s correlation coefficient of the normalized value. Range:-1≤ r ≤1. Values close to 1 indicate a strong positive correlation, meaning more similarity between samples. **(b)** Hierarchical clustering showing the relationships between samples based on the similarity of their expression profiles in the experimental conditions (CC_C and CC_HP) using each sample's normalized value. (Distance metric=Euclidean distance, Linkage method=Complete Linkage). **(c)** Multidimensional scaling showing a 2D plot that represents the similarities and differences between samples in the experimental conditions (CC_C and CC_HP) using each sample's normalized value to identify patterns and outliners.

**Supplementary** **Figure S3.** (a) The Pearson’s correlation between RNA-seq data (y-axis) and RT-qPCR data (x-axis) determined for 20 randomly selected DEGs. All expression data values were normalised (log2). RT-qPCR assays utilised two internal standards, GADPH and EF1. Data is presented relative to the reference gene GAPDH. (b) Expression levels of each gene assessed as Fold Change values, with RT-qPCR data plotted on the left axis and RNA-seq data on the right axis.

**Supplementary** **Figure S4.** The co-expression network of functionally enriched genes in response to (Z)-3-HP in CC plants exhibits significant regulatory effects. A schematic representation of the potential modular organisation of genes involved in subsequent up-regulated transcriptomic events includes secondary metabolite biosynthesis, plant redox balance, plant defence response, and hormone-mediated plant immune response. The edges represent both functional and physical protein associations, with coloured lines indicating the type of interaction evidence. Known interactions are depicted in blue (derived from curated databases) and fuchsia (experimentally determined), while predicted interactions are represented by green (gene neighbourhood), red (gene fusions), and blue (gene co-occurrence) lines.

**Supplementary Table S1.**


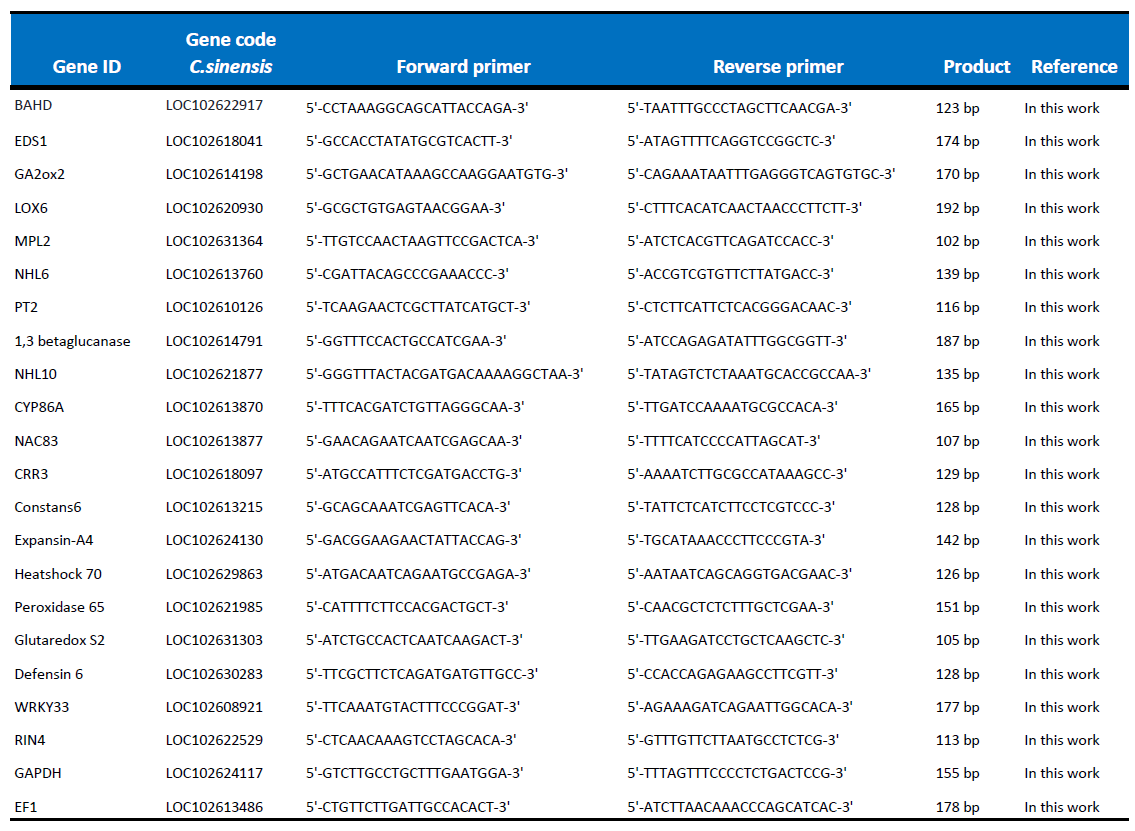


**Supplementary Figure S1**

**
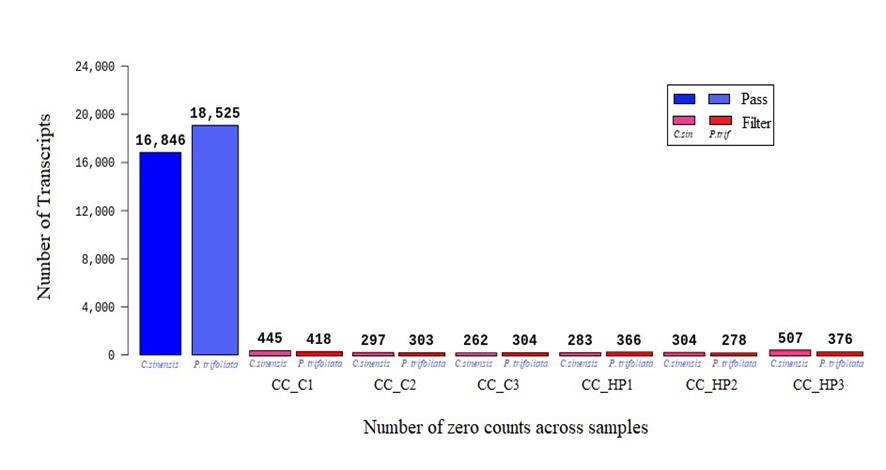
**

**Supplementary Figure S****2**

**
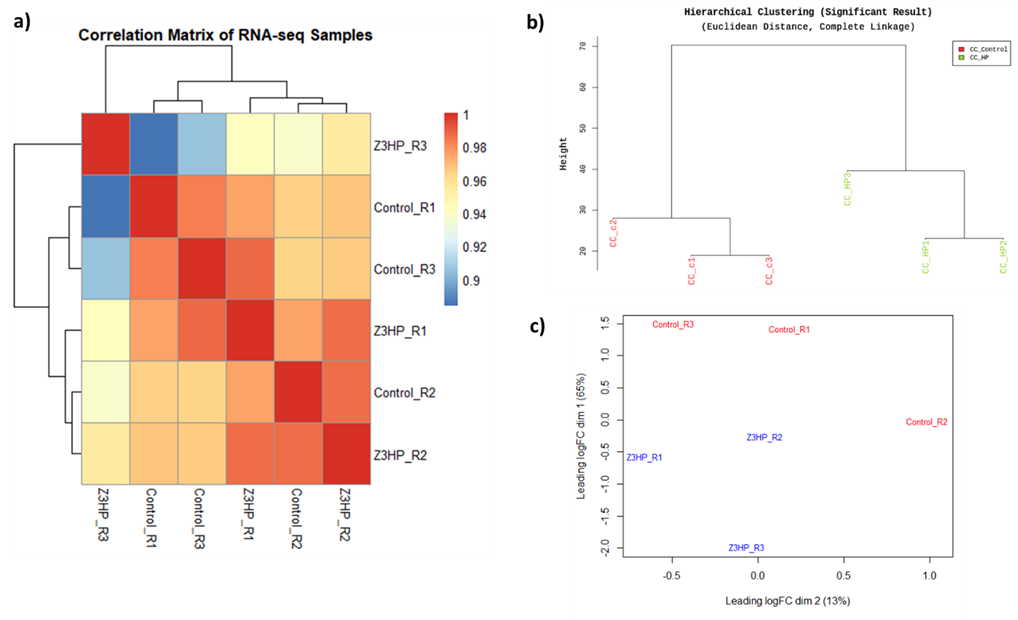
**

**Supplementary Figure S3**

**
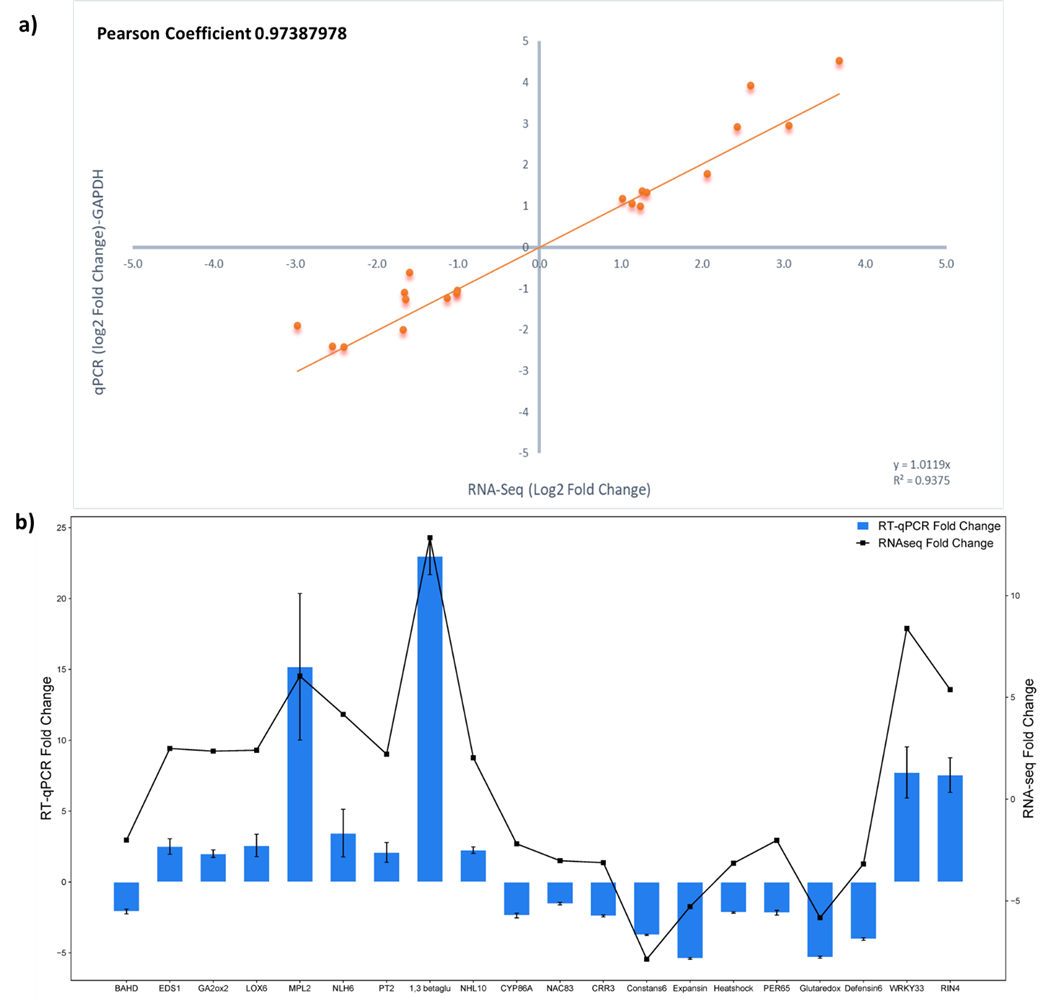
**

**Supplementary Figure S4**

**
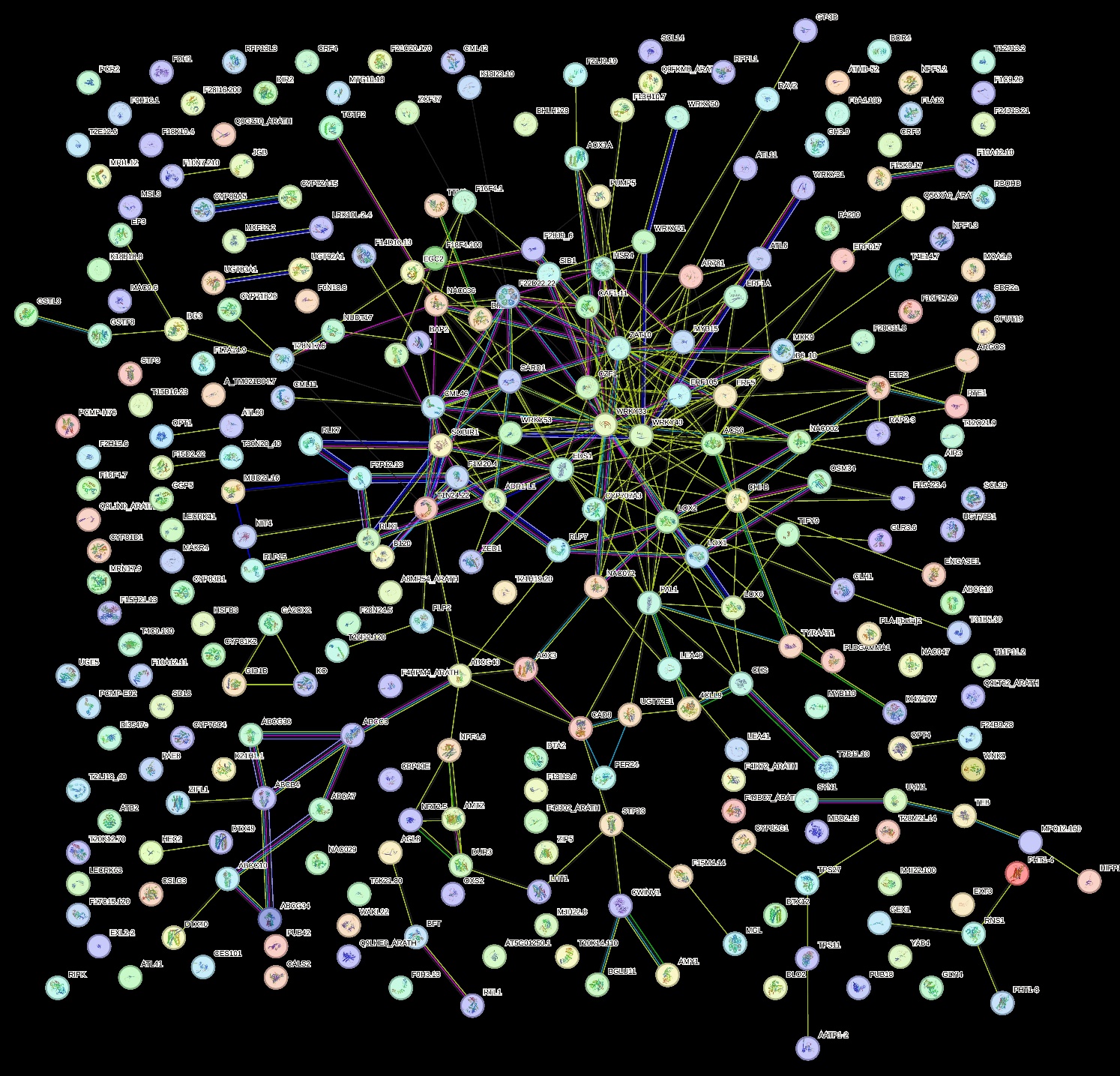
**
